# A calcium-binding protein–sigV regulatory circuit modulates stress adaptation and persistence in *Caulobacter vibrioides*

**DOI:** 10.1007/s00203-026-04894-7

**Published:** 2026-05-13

**Authors:** Elaine Luzia dos Santos, Rita de Cássia Garcia Simão

**Affiliations:** 1 Ministério da Previdência Social, Perícia Médica Federal, Guarapuava, PR Brazil; 2 Universidade Estadual do Oeste do Paraná, Rua Universitária, 1619, 85.819-110 Cascavel, PR Brazil

**Keywords:** *Caulobacter*, Calcium-binding protein, EF-hand, Stress response, ECF sigma factor, Aquatic bacterium

## Abstract

Calcium (Ca²⁺) acts as a second messenger in bacterial stress responses. In *Caulobacter vibrioides*, the *cabp* gene encodes a previously uncharacterized calcium-binding protein (CaBP). In silico analysis predicted a 133-aa protein with three EF-hand motifs and an N-terminal Sec signal peptide. Consistently, CaBP exhibited a Ca²⁺-dependent mobility shift and was localized to the periplasm in a SecA-dependent manner. Transcriptional analyses revealed a non-canonical organization in which the *sigV* promoter lies within the *cabp* coding sequence, although both genes are independently transcribed. Deletion of *cabp* increased biomass and stationary-phase survival. Expression analyses showed that SigmaV modulates *cabp*, while CaBP positively affects *sigV* expression, forming a Ca²⁺ and pH-responsive regulatory loop. Both genes were induced by acid stress and calcium limitation. The mutant displayed altered cell asymmetry and increased extracellular matrix production. These findings identify for the first time that CaBP as a periplasmic EF-hand calcium sensor linking ionic and pH signals to SigmaV-dependent stress regulation.

**Supplementary Information:**

The online version contains supplementary material available at 10.1007/s00203-026-04894-7.

## Introduction

*Caulobacter vibrioides* (*C. crescentus* heterotype) is a Gram-negative alpha proteobacterium widely used as a model for studies of gene regulation and cell differentiation. Its life cycle involves asymmetric division, giving rise to motile, flagellated cells, sessile, stalk-attached cells (holdfast), and pre-divisional cells that regenerate both forms, ensuring replication and coordinated cell cycle progression (Barrows and Goley 2023). Differentiation events in *C. vibrioides* are spatially and temporally coordinated by complex regulatory networks involving hundreds of genes and reflect its adaptation to oligotrophic environments (Jenal 2000; Marks et al. 2010; Xu et al. 2020). Among environmental factors, calcium stands out as a multifunctional regulator, participating in the assembly of extracellular structures, protein stabilization, and indirect modulation of intracellular signaling pathways (Walker et al. 1994).

Recent evidence shows that bacterial proteins containing EF-hand motifs can act as Ca²⁺ sensors, adjusting gene expression to ionic variations—as observed for the *Pseudomonas aeruginosa* EfhP protein, which regulates iron uptake and virulence genes according to Ca²⁺ levels (Burch-Konda et al. 2024). Data also highlight the functional expansion of calcium-binding proteins in prokaryotes, which comprise an “ion-sensing arsenal” associated with environmental adaptation (Agaras et al. 2023). Calcium acts as a ubiquitous second messenger that modulates microbial physiology and adaptation to environmental stresses. Studies demonstrate calcium-mediated regulatory mechanisms in bacteria, ranging from biofilm formation and metabolic regulation to ecological processes in photosynthetic sulfur (Pu et al. 2020; Wu et al. 2021; Madigan et al. 2024).

In *C. vibrioides*, the holdfast—a polar adhesive structure composed of polymers of N-acetyl-D-glucosamine, peptides, and DNA—ensures attachment, mechanical strength, and biofilm formation (Fiebig 2019). The HfaA–D proteins participate in polymer export and anchoring, with possible indirect dependence on calcium during assembly (Kurtz and Smith 1994). Furthermore, the surface protein RsaA forms the paracrystalline layer (S-layer), whose assembly and stability require Ca²⁺ to establish ionic bridges between RsaA and lipopolysaccharide (Gilchrist et al. 1992; Smit et al. 1992). Studies have revealed that S-layer biogenesis is coordinated with the cell cycle and sensitive to the availability of metal ions, including calcium, demonstrating the integration of structural and physiological control of the cell surface (Herdman et al. 2022, 2024).

In addition to these structures, *C. vibrioides* expresses calcium-binding proteins such as caulolins and the Hfa components of the holdfast. These caulolins, presumably periplasmic, bind Ca²⁺ with micromolar affinity and acquire an ordered conformation only after this binding, in a structural activation mechanism like that of eukaryotic calmodulins (Jobby and Sharma 2007).

Ca²⁺-dependent conformational changes have also been described in bacterial β-γ-crystallin domains, suggesting evolutionary conservation of structural strategies mediated by divalent ions (Srivastava et al. 2010). Although the holdfast and S-layer exert distinct functions, their coordinated assembly is essential for cellular integrity and differentiation, being influenced by both Ca²⁺ availability and cell cycle-coupled transcriptional regulation (Domínguez et al. 2015; Herdman et al. 2024). The *C. vibrioides* genome contains at least five genes encoding proteins with characteristic EF-hand domains (Marks et al. 2010). These CaBPs participate in ion homeostasis and stress response, but Ca²⁺-dependent signaling mechanisms in prokaryotes remain poorly understood (Domínguez et al. 2015; Burch-Konda et al. 2024).

In eukaryotes, EF-hand–CREC–Calumenin-like proteins act as luminal chaperones involved in calcium homeostasis and Ca²⁺-dependent protein trafficking (Mazzorana et al. 2016). Although EF-hand motifs are known in bacteria, analogous CREC–Calumenin-like proteins have not yet been characterized, indicating a possible new functional group with a predominantly structural and regulatory role.

To date, there has been no established relationship between CaBP proteins and ECF (Extracytoplasmic Function) Sigma-type factors, responsible for controlling the transcription of genes linked to cell envelope integrity and stress response. In *C. vibrioides*, the Sigma 54 (RpoN) and Sigma 70 (RpoD) factors act under physiological conditions (Malakooti and Ely 1995; Wu et al. 1997), while Sigma 32 (RpoH) responds to heat shock (Reisenauer et al. 1996) and 13 other ECFs modulate specific responses (Marks et al. 2010). Among them, SigmaE–ChrR and Sigma F stand out, involved in the response to heavy metals and oxidative stress (Alvarez-Martinez et al. 2006, 2007; Kohler et al. 2012). The Sigma T factor, considered the main global regulator of the general stress response, acts with PhyR and NepR controlling a large part of the ECF regulon (Lourenço et al. 2011). A recent review (Mascher 2025) emphasizes the central and evolutionarily diverse role of Sigma ECFs in environmental bacteria, highlighting their importance in physiological adaptation.

Although canonical calmodulins are absent in bacteria, calmodulin-like proteins containing EF-hand motifs have been described in several prokaryotic systems based on both sequence and immunological evidence. Notably, genome-wide analyses have identified multiple EF-hand proteins in *C. vibrioides*, including CCNA_02838/CC_2752, which belongs to a group of multi-EF-hand proteins with potential roles in stress response and calcium-dependent regulation (Michiels et al. 2002). In the present work, we characterize for the first time the *cabp* gene, which encodes a calcium-binding protein (CaBP) containing EF-hand–CREC–Calumenin-like motifs, and investigates its functional relationship with the previously undescribed extracytoplasmic sigma factor SigmaV (SigV). The study analyzes the gene organization and regulatory context of these genes, elucidating aspects of their calcium-dependent expression and proposing a model that links CaBP to ion homeostasis and the adaptation of *C. vibrioides* to variable environmental conditions.

## Materials and methods

### Bacterial strains and growth conditions

*Escherichia coli* strains were grown at 37 ° C in Luria–Bertani medium (LB; tryptone 10 g L⁻¹, yeast extract 5 g L⁻¹ and NaCl 10 g L⁻¹, pH 7.5) supplemented with the appropriate antibiotics: ampicillin (100 µg mL⁻¹), chloramphenicol (30 µg mL⁻¹) or tetracycline (12.5 µg mL⁻¹). *C. vibrioides* (strain NA1000) was grown at 30 ° C in PYE complex medium (peptone 2 g L⁻¹, yeast extract 1 g L⁻¹, MgSO₄ 7 H₂O 0.2 g L⁻¹ and 0.5 mM CaCl₂) or in M2G minimal medium (8 mM Mg₂HPO₄, 8 mM KH₂PO₄, 9 mM NH₄Cl, 10 µM FeSO₄, 2 mM MgSO₄, 0.5 mM CaCl₂ and 0.2% glucose (v/v) or 0.3% xylose (v/v) supplemented, when necessary, with nalidixic acid (20 µg mL⁻¹), tetracycline (2 µg mL⁻¹) and chloramphenicol (1 µg mL⁻¹). Plasmids and primers used in the present work are discriminated in the Table 1.

**Table 1:** Plasmids and primers used

Plasmids/Primers	Properties/Sequences (5’- 3’)	Source
pAS22	Gene expression vector for *C. vibrioides*; P*xyl*X promoter, ori T, CmR, expression depending on xylose addition (0.3% v/v)	(Meisenzahl et al. 1997)
pAS22-CaBP	pAS22 + gene *cabp*	This work
placZ290	Vector for transcription fusion	(Gober and Shapiro 1992)
ProCaBP	Transcriptional fusion of the *cabp* promoter with lacZ	This work
pUCBM21-cabp	Coding region of the cabp gene cloned in pUCBM21, ApR	This work
pJS14cabp	*Eco*RI/*Bam*HI fragment containing the coding region of the cabp gene, the gene is expressed under the control of the *lacZ* promoter in a constitutive manner	This work
cabp 3’	tatggatcccaaaactcagctcgccgc	This work
cabp 5’	tatgaattcgtccattggcgcgacgga	This work
Pro*cabp*3’	tatggatcccatgatcgttcatctccatc	This work
Pro*cabp*5	tatgaattcgactggaggcggcctggc	This work
sigV-3	tatggatccccttcatcacgcttcgcc	This work
sigV-5	tatgaattcgcggcgagctgagttttg	This work
*cabpXho*3’	ttatctcgaggacgctgacaaggatcgc	This work
*cabpXho5’*	ttatctcgagggtgatcttgccgtccctg	This work
cabp*Forw*	tatgaattcgtccattggcgcgacgga	This work
cabp*rev*	ttatctcgagggtgatcttgccgtccctg	This work
sigVForw	atgaattcgcggcgagctgagttttg	This work
sigV del 5’	ttatctcgagattcagcgccacgcggtg	This work

## Cloning of the *cabp* gene

The *cabp* gene (CCNA_02838) and its promoter region were amplified by PCR from the *C. vibrioides* NA1000 genome using the specific oligonucleotides (Table 1). The primers were designed based on the published genomic sequence (Marks et al. 2010) and included artificial restriction sites for cloning into the pUCBM21 vector. The amplified fragment was subsequently subcloned into the vectors pUJ14 (*Eco*RI/*Bam*HI) generating the pRSCaBP strain, used to complement the calcium binding protein mutant (ΔCaBP-C strain). All constructs and recombinant strains are described in Table 2.

**Table 2:** Bacterial strains used in the present work

Strains	Genotype/Phenotype	Source
*E. coli*		
DH10B	F– Δ(*mrr-hsdRMS-mcrBC*)φ80d*lacZ* ΔM15 *mcrA* Δ*lacX74 endA1 recA1deoR* Δ*(ara*,* leu)7697 araD139 galU nupG rpsL*	Gibco BRL
S17-1	F– lambda (–) *thi pro recA hsdR*– *hsdM*+ RP4 derivative integrated into the chromosome with Tet::Mu, Km::Tn*7*	(Simon et al. 1983)
*C. vibrioides*		
WT	Synchronizable wild-type strain NA1000 derived from *C. vibrioides* lineage CB15	(Evinger and Agabian 1977)
CaBP^++^	Line NA1000 containing pAS22-cabp, expresses the gene in the presence of xylose	This work.
ΔCaBP	NA1000 with 100 bp in-frame deletion of the cabp gene (CCNA_02838)	This work.
ΔCaBP-C	ΔCaBP complemented with the pJS14-cabo vector	This work.
NA1000-ProCaBP	NA1000 carrying the transcriptional fusion between lacZ and the cabp promoter region	This work.
NA1000-*placZ290*	NA1000 containing the placZ plasmid without transcriptional fusion	This work.
CaBP^++^ ProCaBP	CaBP + + carrying the transcriptional fusion between lacZ and the cabp promoter region	This work.
CaBP^++^*placZ290*	CaBP + + containing the placZ plasmid without transcriptional fusion	This work.
ΔCaBP - ProCaBP	ΔCaBP carrying the transcriptional fusion between lacZ and the cabp promoter region	This work.
ΔCaBP *placZ290*	ΔCaBP containing the placZ plasmid without transcriptional fusion	This work.
PRSCaBP	ΔCaBP containing the pJS14-*cabp*	This work
ΔsecA	LS416 mutant strain null for the *secA* gene (secretion/translocation)	(Kang and Shapiro 1994)
ΔSigT	Strain ML161 null mutant for the _*sigT*_ gene (CC 3475)	(Alvarez-Martinez et al. 2007)
ΔSigT++	Strain PRL480 NA1000 that overexpresses *sigT*	(Alvarez-Martinez et al. 2007)
ΔSigV	SG100 gene null mutant strain (CCNA_02837)	(Lourenço and Gomes 2009)
SigV++	Strain PRL180: NA1000 that overexpresses *sigV*	(Lourenço and Gomes 2009)
ΔSigV/ProCaBP	SG100 gene null mutant strain (CCNA_02837) containing the *cabp* gene promoter cloned into lacZ290 (Transcriptional fusion)	This work.
SigV + + ProCaBP	PRL180:NA1000 strain overexpressing *sigV* containing the *cabp* gene promoter cloned into lacZ290 (Transcriptional fusion)	This work.

## Construction of the ΔCaBP mutant

To generate the ΔCaBP null mutant, the *cabp* gene (CCNA_02838) previously cloned in pUCBM21 was used as a template in PCR reactions with two pairs of flanking primers containing *Xho*I restriction sites. These fragments were annealed to internally delete 81 bp corresponding to one of the EF-hand Ca²⁺-binding domains, preserving the reading frames of adjacent genes. The deleted fragment was cloned into the suicide vector pNPTS138 (kanamycin resistance; *sacB* gene), which does not replicate in *C. vibrioides*. To generate the ΔCaBP mutant, the internal 81 bp fragment of the *cabp* coding region deleted was replaced by a spectinomycin resistance cassette. The deletion was designed to maintain the downstream reading frame to preserve regulatory elements associated with the sigV gene located within the *cabp* coding region. The pNPTS-Δcabp construct was introduced into the donor strain *E. coli* S17-1 for conjugation with *C. vibrioides* NA1000. Kanamycin-resistant exconjugants were selected on PYE medium and confirmed by Southern blot, detecting copies of the native gene and the deleted version (81 bp shorter). The second homologous recombination was induced by counterselection on PYE medium containing 3% sucrose, and kanamycin-sensitive and spectinomycin resistant clones were isolated. (Figure S1). Vector loss and the exclusive presence of the deleted copy were verified by repeat PCR and Southern blot analysis. Three independent clones (ΔCaBP 2, 4, and 6) showed the expected pattern, confirming that *cabp* is not essential for growth under laboratory conditions.

### RNA extraction and 5’RACE

Cultures of the WT, ΔCaBP, ΔSigV, SigV++, and CaBP + + strains were grown at 30 ° C to log phase (O.D.₆₀₀ ≈ 0.6–1.0). For CaBP++, induction was achieved by the addition of 0.3% (v/v) xylose. For 5′ RACE assays, cultures were subjected to calcium limitation with EGTA. Total RNA was extracted using Trizol™ (Thermo Fisher^®^) after lysis in DEPC-treated sodium acetate/EDTA/SDS buffer. RNA was resuspended in DEPC-treated Milli-Q water and stored at − 40° C. Integrity was verified by electrophoresis on a 1.5% agarose gel containing formaldehyde, and concentrations were adjusted based on absorbance at 260 nm. The transcription initiation of the *cabp* gene was determined using the 5’ RACE kit (Roche^®^), according to the manufacturer’s instructions. For cDNA synthesis, 2 µg of total RNA was used as a template, employing a gene-specific oligonucleotide (*cabp*Xho5’) that hybridizes directly to the mRNA of interest. After cDNA purification, a poly(A) tail was added to its 3’ end through enzymatic transfer of adenine residues. The polyadenylated cDNA was then used as a template in a PCR amplification reaction in the presence of a gene-specific primer (P*cabp*3’), located closer to the poly(A) end than the oligonucleotide used in the previous step, and an anchor primer consisting of 16 thymine nucleotides followed by a defined anchor sequence. The product of this first amplification was subjected to a second PCR reaction (nested PCR) using a primer complementary to the anchor sequence and a second gene-specific primer (P*cabp*3’) that binds even closer to the poly(A) region. The amplified product was then cloned into the pGEM-T Easy vector (Promega^®^), and five independent clones were sequenced. Sequence analysis identified the nucleotide immediately adjacent to the thymine region, corresponding to the exact transcription start site of the *cabp* gene.

### qPCR and qualitative RT-PCR

Reverse transcription was performed with the SuperScript™ III Reverse Transcriptase/Platinum Taq Mix kit (Invitrogen^®^) according to the manufacturer’s instructions. Equivalent amounts of total RNA were used in all samples. Reactions were conducted at 50 ° C for 30 min, followed by inactivation at 94 ° C for 2 min. Amplification was performed with 40 cycles (94 ° C 30 s; 55 ° C 45 s; 68 ° C 60 s), followed by final extension at 68 ° C for 5 min. Negative controls without reverse transcriptase confirmed the absence of contaminating DNA. The products were analyzed on a 1.5% (w/v) agarose gel in 1× TBE buffer.

### Western blot immunodetection

Cells were grown in PYE 30 ° C until initial log phase, after that, cells were submitted to 37 ° C and harvested after 1–3 h of growth (depletion of SecA protein is obtained at 37 ° C). Cells were collected and centrifuged at 12,000 × g, 3 min. Cytosolic and periplasmic protein extracts from different *C. vibrioides* strains were prepared according to the method described by Steinman and Ely (1990). After that, different extract were resuspended in buffer O (100 mM Tris-HCl pH 6.8; 4% SDS; 0.2% bromophenol blue; 20% glycerol). Samples were boiled, centrifuged, and loaded (20 µL) onto 15% SDS-PAGE (Laemmli 1970). Proteins were transferred to nitrocellulose membranes (Towbin et al. 1979) and blocked with TBS + 5% milk. The membranes were incubated for 10 h with anti-calmodulin antibody from *Blastocladiella emersonii* (Simão and Gomes 2001) (1:200, TBS-Triton tween at 0.01% v/v) and anti GroEL (1:100) anti-sera from *C. crescentus* (Baldini et al. 1998) for cytosolic proteins control, washed and incubated with anti-IgG secondary antibody conjugated to alkaline phosphatase (Sigma^®^). Development was done with NBT/BCIP in Tris-HCl buffer pH 9.0.

### Complementation of the ΔCaBP mutant

The ΔCaBP strain was complemented by conjugation with *E. coli* S17-1 containing the pUJ14*cabp* plasmid. Conjugation occurred in solid PYE at 30 ° C for 48 h. Exconjugants (ΔCaBP-C) were selected in PYE + chloramphenicol (1 µg mL⁻¹) + nalidixic acid (50 µg mL⁻¹), which inhibits *E. coli* growth. ΔCaBP-C strain was used as a control in different assay in the present report.

### Growth curves and cell viability tests after acid shock

The NA1000 (WT), ΔCaBP, and ΔCaBP-C strains were cultured in PYE at 30 ° C, under agitation at 120 rpm, and monitored by optical density (OD = 600 nm). Samples were collected at regular intervals to estimate growth and viability by survival assays. Bacterial cell survival was measured by counting colony-forming units (CFU) of the samples grown and diluted serially (10⁻^1^ to 10⁻⁶). The different dilutions were plated in duplicate on solid PYE and incubated at 30 ° C for 48 h. CFU were counted after 48 h of growth and the data expressed graphically in CFU. Growth curves and survival assays were performed in biological triplicate. The evaluation of survival to acid shock (10mM HCl) was performed with cells in the logarithmic growth phase, which were subjected to pH 4 for different times. The ability of cells to recover from acid shock was measured by transferring them to standard culture medium and monitoring their growth for up to 48 h. Cell growth was monitored by spectrophotometry at an OD of 600 nm.

### Transcription fusion and promoter activity assays

The *cabp* gene promoter activity was analyzed by fusion with *lacZ* in the pPlacZ290 (Gober and Shapiro 1992) vector, generating the ProCaBP construct. The regulatory region was cloned upstream of *lacZ* and the constructs introduced by conjugation into *C. vibrioides*. The strains were grown in PYE under different stress conditions (85 mM NaCl; 150 mM sucrose; 15% (v/v) ethanol; 0.01% (v/v) toluene; Ca^2+^ ranging from 50 mM (control condition) to 250 mM (5-fold higher); 1 mM EGTA; pH 4. β-galactosidase activity was determined and expressed in Miller Units (Griffith and Wolf 2002).

### Morphological analysis by fluorescence microscopy

For morphological and cellular organization analysis, samples of *C. vibrioides* strains NA1000 (wild-type), ΔCaBP, and ΔCaBP-C were grown in PYE medium at 30 ° C under moderate shaking (120 rpm) until stationary phase. Cells were collected by centrifugation (5000 × g, 5 min) and washed twice with 0.1 M phosphate buffer (pH 7.2). For cell membrane labeling, the lipophilic dye FM 5–95 (Invitrogene^®^) was used, added at a final concentration of 5 µg mL⁻¹. Samples were incubated for 5 min at room temperature, washed twice in phosphate buffer, and observed immediately. The dye was excited at 561 nm and detected between 600 and 630 nm. Images were obtained using a Nikon TE 300 inverted fluorescence microscope equipped with a high-resolution digital camera and FM 5–95 filters. Micrographs were analyzed using ImageJ v1.54p software (Schroeder et al. 2021) for contrast adjustment, cell area measurement, and channel overlay. Assays were performed in experimental duplicates and with at least three replicates.

### Scanning electron microscopy (SEM)

To prepare samples for scanning electron microscopy, *C. vibrioides* cells were grown in tissue culture plates containing PYE medium at 30 ° C, under gentle shaking (60 rpm), until they reached the stationary phase. The optical density (OD₆₀₀) of the cultures was monitored, and aliquots were diluted in PYE medium until OD₆₀₀ = 0.1, being incubated under the same conditions in tissue culture plate until reaching values ​​between 0.8 and 1.0, corresponding to the beginning of the stationary phase (Figure S2). The adhered cells were washed with 1 mL of 0.1 M phosphate buffer (pH 7.2) for 1 min at 30 ° C. This procedure was repeated three times. Then, the cells were fixed in a freshly prepared solution containing 2.5% glutaraldehyde and 2% paraformaldehyde in 0.1 M phosphate buffer (pH 7.2) for 2 h at 30 ° C, under gentle shaking (20 rpm). After primary fixation, the mini-culture plates were washed three times with 0.1 M phosphate buffer (pH 7.2) and subjected to post-fixation in 1% osmium tetraoxide (OsO₄) prepared in the same buffer for 1 h at 30 ° C. The samples were washed again, dehydrated in an increasing series of ethanol (30%, 50%, 70%, 90% and 100%) and dried under laminar flow for 2 h at 30 ° C. Subsequently, mini-plate fragments (0.5 × 0.5 cm) were cut with a sterile metal knife, mounted on sample holders (stubs) containing double-sided carbon tape, and subjected to sputtering with a thin layer of gold. Observations were performed using a Tescan^®^ Vega3 scanning electron microscope, installed at the Federal University of Paraná (UFPR, Palotina, Brazil). Micrographs were acquired at magnifications ranging from 5,000× to 50,000×, using a secondary electron (SE) detector. SEM analysis was used to examine cellular morphology and structure, as well as the presence of abundant extracellular material surrounding mutant cells, without direct assessment of adhesive function, in comparison with the parental and complemented strains. All assays were conducted in at least three independent biological replicates performed in duplicate.

### AI software and tools

Gene and protein sequences were obtained from the Kyoto Encyclopedia of Genes and Genomes (KEGG) database (Kanehisa et al. 2021). The restriction map for cloning was constructed using the Restriction Map algorithms available in the Bioinformatics Sequence Manipulation Suite (Stothard 2000). Specific primers were designed using Primer Express software (Applied Biosystems^®^). The predicted three-dimensional structures of the SigV and CaBP proteins were generated by artificial intelligence-based protein folding modeling using AlphaFold (Jumper et al. 2021). Signal sequence prediction was carried out using Phobius (Käll et al. 2004) and SignalIP-6.0 (Teufel et al. 2022). RT-qPCR data were processed using StepOne/StepOnePlus v2.3 software (Thermo Fisher Scientific^®^), and statistical analyses of variance (ANOVA) were performed using Microsoft Excel v16.100.4/2025 (Microsoft Corporation^®^).

## Results

### Analysis of gene structure and predicted protein

This study evaluated the role of the *cabp* gene (CCNA_02838) from *C. vibrioides*. The gene has no defined function in the bacterium and, to our knowledge, has not been studied in any other prokaryotic system, although GenBak analysis indicates it is conserved in numerous bacteria. The *cabp* gene has 402 nucleotides and encodes a 133-amino acid protein. The highest percentage of identity/similarity of *cabp* from *C. vibrioides* was observed in the related bacterium *Caulobacter segnis* (54%/63%), followed by dozens of other bacteria of the same genus, although there is still low identity/similarity with other EF-hands from other alpha-Proteobacteria such as *Phenylobacterium sp* (45%/55%), *Pseudomonadota sp* (36%/50%), and *Salipiger sp (*40%/57%). The predicted amino acid sequence from the *C. vibrioides cabp* gene reveals three calcium-binding motifs typical of the ubiquitous eukaryotic protein family called EF-Hand-CREC-Calumenin-like.

**Fig. 1:**
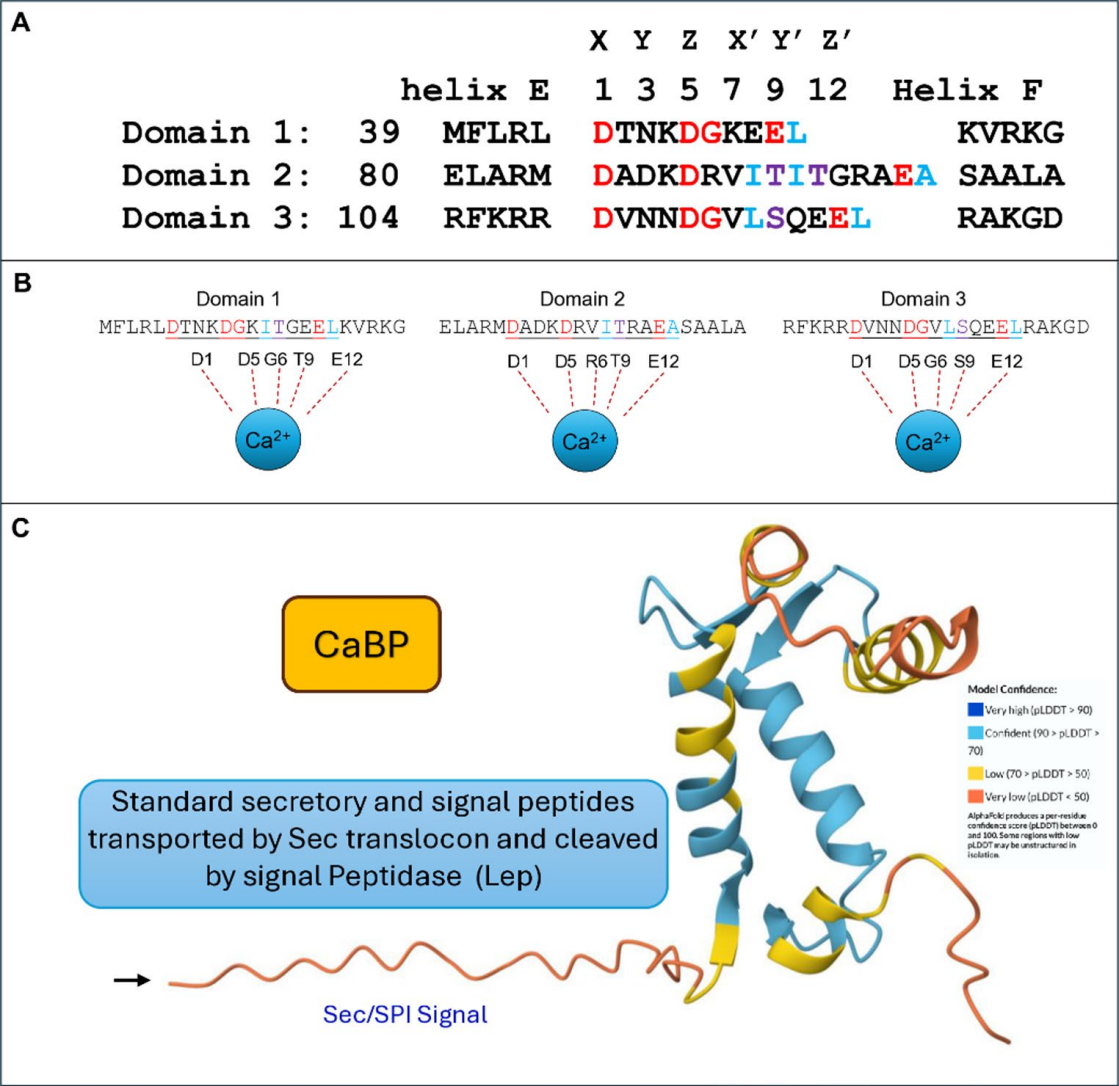
Consensus EF-hand motifs and predicted 3D structure of *C. vibrioides* CaBP. **A** Alignment of the three EF-hand domains showing helices E and F and the Ca²⁺-binding loop. The Prosite for -EF-hand signature (Swiss Institute of Bioinformatics, SIB) includes the 12 loop residues plus the fourth residue of helix F. Conserved Ca²⁺-coordinating residues at positions X, Y, Z, –Y, –X, and –Z are indicated; their carboxyl and carbonyl oxygens form a pentagonal bipyramidal coordination geometry illustrated in (**B**). Highly conserved residues are highlighted in red, with conservative substitutions (D↔E, S↔T) shown in purple. **C** AlphaFold-predicted 3D structure of CaBP showing the N-terminal signal peptide (black arrow), characteristic of periplasmic localization. AlphaFold model confidence values are indicated by color scale. The predicted structure shows high confidence (pLDDT > 70) in the EF-Hand regions

Indeed, in silico analysis of the *cabp* gene and the predicted protein indicated that the *C. vibrioides* CaBP protein exhibits three EF-hand motifs with conservation of typical Ca²⁺ coordinating residues (Fig. 1A).

These motifs include 12 residues with conservation of essential coordinating positions in these domains (Asp 1, Asp 5, and Glu 12), indicating a likely Ca²⁺-binding capacity (Fig. 1A). Although motifs 1 and 3 preserve the glycine at position 6, which is necessary for the loop geometry, motif 2 presents an atypical substitution (Arg 6), which may alter the loop conformation and reduce its affinity for Ca²⁺. Therefore, it is likely that there are heterogeneous affinities for Ca²⁺ ions between the domains of the bacterial CaBP. No bacterial homologs with experimentally characterized function were identified in GenBank or PDB, indicating that CaBP belongs to a functionally unannotated clade, which warrants further biophysical studies. Modeling of the predicted protein in AlphaFold (Jumper et al. 2021) also revealed a 21-aa N-terminal signal peptide that, according to the predictor (Käll et al. 2004) (Figure S3), is a typical signal of the Sec/SPI secretion pathway (Teufel et al. 2022), suggesting a periplasmic and/or extracellular localization for the CaBP of *C. vibrioides* (Fig. 1B).

### Characterization of the promoter region and construction of a Δ-CaBP null mutant

**Fig. 2:**
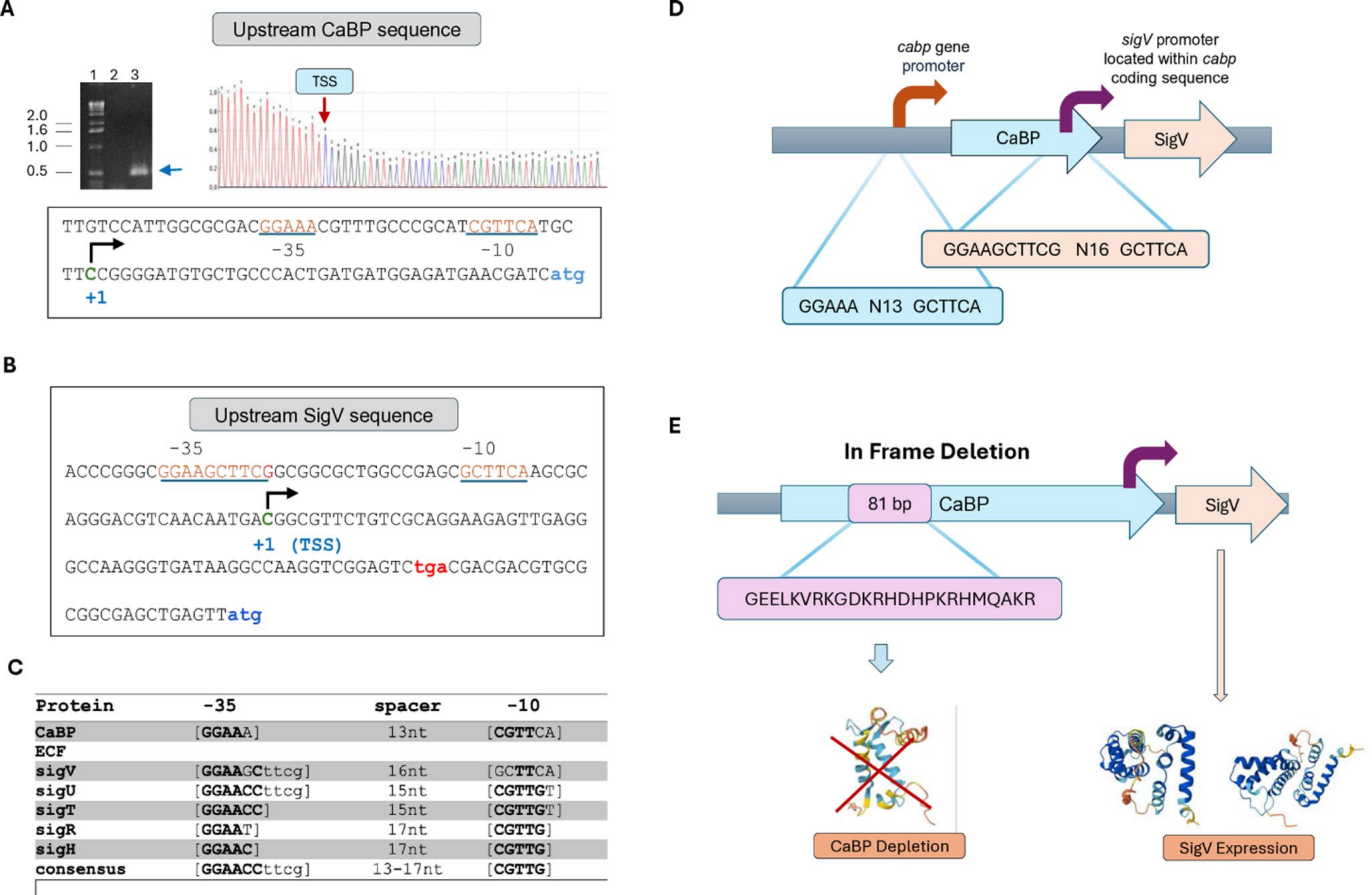
Transcriptional organization and genomic architecture of the *cabp–sigV* locus in *C. vibrioides*. **A** Identification of the *cabp* transcript by RT-PCR under calcium-chelating conditions. Total RNA from *C. vibrioides* cells grown in the presence of 1 mM EGTA was used for RT-PCR amplification of the *cabp* transcript. PCR products were analyzed by agarose gel electrophoresis. Lane 1, molecular weight marker; Lane 2, negative control (no template); Lane 3, RT-PCR product obtained from cells grown in the presence of EGTA. EGTA chelates Ca²⁺ ions, reducing the availability of free calcium in the medium and inducing *cabp* gene expression. 5′ RACE mapping identified the transcription start site (TSS) of the *cabp* gene at 40 nt upstream of the ATG start codon (blue). The − 10 and − 35 promoter motifs are underlined in red. **B** The *sigV* regulatory region harbors an intragenic promoter within the *cabp* coding sequence, whose transcription start site (TSS) was defined by transcriptomic analyses (Bharmal et al. 2020) and is positioned 87 nt upstream of the *sigV* start codon and 58 nt upstream of the *cabp* stop codon. The − 10 and − 35 boxes are underlined in red. **C** Comparison between the *cabp* promoter consensus and ECF-type sigma factor promoter motifs, including *sigV*. **D** Schematic representation of the *cabp–sigV* genomic arrangement. **E** Sequence corresponding to the *cabp* in-frame deletion, located downstream and outside the *sigV* promoter region. In the null mutant of the *cabp* gene, the in-frame deletion carried out does not affect the expression of the *sigV* gene downstream of the *cabp* gene, but the deletion ensures that the gene is not expressed and the CaBP protein is not synthesized. Therefore, there is no functional intergenic region separating these genes

In the genomic context, the *cabp* gene is located adjacent to the ORF CCNA_02837, whose predicted product corresponds to an uncharacterized sigma factor of the extracytoplasmic function (ECF) family. In this study, this gene was designated *sigV*, and its predicted protein as SigmaV (SigV). Qualitative RT-PCR analyses showed that *cabp* expression increases under calcium depletion conditions in the wild-type strain NA1000 (WT) (Fig. 2A). To characterize the promoter region of *cabp*, a 5′ RACE assay was performed using total RNA extracted from wild-type cells exposed to 1 mM EGTA for 1 h (Fig. 2A). The transcription start site (+ 1) of *cabp* was mapped 40 nucleotides upstream of the translation start codon (ATG). Based on this experimentally defined TSS, the − 35 and − 10 promoter elements were identified within the 5′ non-coding region, with sequences − 35 [GGAAA], a 13-nt spacer, and − 10 [CGTTCA] (Fig. 2C).

The transcription start site of *sigV* has been previously defined by transcriptomic analyses (Bharmal et al. 2020; Lasker et al. 2016). These data indicate that the *sigV* TSS is located 87 nucleotides upstream of its translation start codon and within the 3′ terminal region of the *cabp* coding sequence, specifically 58 nucleotides upstream of the stop codon. Accordingly, *sigV* is driven by an intragenic promoter embedded within the *cabp* coding region, with predicted − 35 [GGAAGCTTCG], a 16-nt spacer, and − 10 [GCTTCA] motifs (Fig. 2C).

This genomic arrangement indicates that *cabp* and *sigV* possess independent promoters despite their overlapping organization and the absence of a functional intergenic region. Consistent with this architecture, both genes are transcribed as monocistronic units rather than as part of an operon.

To evaluate the functional role of *cabp* in *C. vibrioides*, the gene was cloned and used to construct a mutant strain by in-frame deletion through homologous recombination, as described in the methodology (Figure S3; Fig. 2D). This strategy removed 81 base pairs (corresponding to 27 amino acids; Fig. 2E) while preserving the reading frame of downstream sequences and maintaining the integrity of the intragenic *sigV* promoter region, ensuring that *sigV* transcription is not disrupted.

### Characterization of the null mutant Δ-CaBP regarding growth and cellular localization

**Fig. 3:**
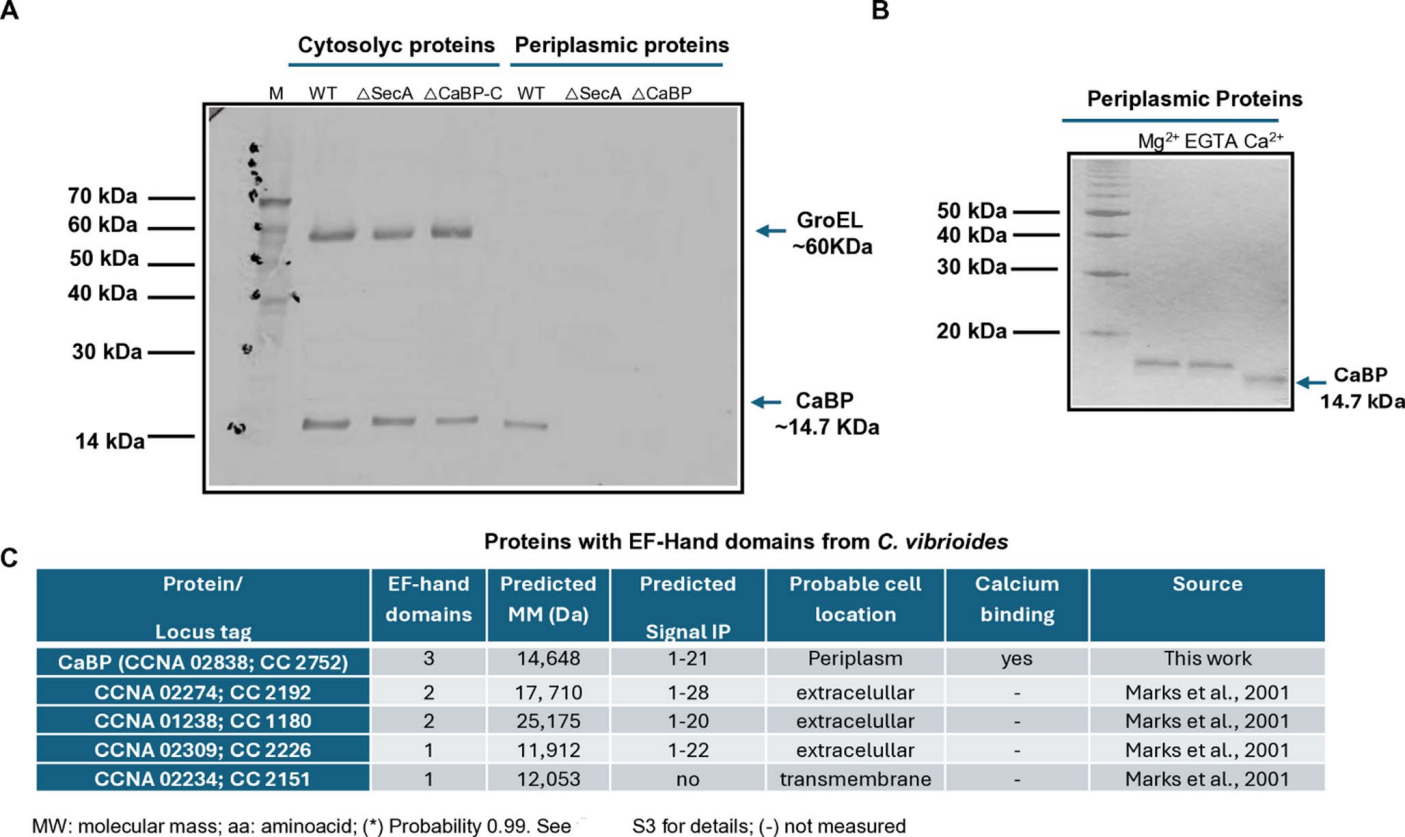
**A** Western blot analysis of GroEL (cytosolic) and CaBP (periplasmic) proteins from *C. vibrioides* and Ca²⁺-dependent SDSPAGE mobility shift. A Total and periplasmic protein extracts from WT, ΔCaBP, ΔCaBP-C and Δ*secA* strains were separated on 15% SDS– PAGE and transferred to nitrocellulose membranes. Detection was performed using a polyclonal anti-calmodulin antibody from *B. emersonii* (Simão and Gomes 2001) and anti-GroEL from *C. crescentus* (Baldini et al. 1998) followed by a rabbit anti-IgG conjugated to alkaline phosphatase. Protein–antibody complexes were visualized with NBT/BCIP staining. M: pre-stained molecular weight marker; WT: *C. vibrioides* NA1000 strain; ΔSecA: *secA* mutant strain at 37° C; ΔCaBP-C: *cabp* complemented mutant; ΔCaBP: *cabp* null mutant by in frame deletion. Data represent the mean of three independent experiments; **B** Periplasmic proteins extract from *C. vibrioides* were separated on 15% SDS-PAGE before incubation at 37 °C in the presence of MgCl_2_ (5 mM); EGTA (5 mM) and CaCl_2_ (5 mM) during 20 min at 4 °C. The proteins were resolved by SDSPAGE 15%, transferred for nitrocellulose membrane and incubated with the polyclonal anti-sera anti-calmodulin from *B. emersonii*. M: prestained molecular weight marker. Data represent the mean ± SD from three independent experiments. **C** Predicted traits for the five EF-Hand proteins annotated in the genome of the *C. vibrioides* NA1000 strain (Marks et al. 2010)

To evaluate the effect of the in-frame deletion of part of the *cabp* gene in *C. vibrioides* (Fig. 2E), the presence or depletion of the CaBP protein was assessed in different bacterial strains by Western blot analysis using polyclonal antibodies against GroEL (Baldini et al. 1998) from *C. crescentus* and calmodulin from the aquatic fungus *B. emersonii* (Simão and Gomes 2001), a protein containing four canonical EF-hand calcium-binding domains.Structural predictions obtained using AlphaFold (Jumper et al. 2021), Phobius (Käll et al. 2004) and SignalP-6.0 (Teufel et al. 2022) indicated that the CaBP protein of *C. vibrioides* contains a signal peptide that likely directs the protein to the periplasmic space and/or extracellular environment through the Sec/SPI secretion pathway (Fig. 1C). To experimentally evaluate the cellular localization of CaBP, cytoplasmic and periplasmic protein fractions were prepared from the parental strain NA1000 (WT), the secretion mutant ΔSecA (strain LS416, defective in SecA-dependent secretion at 37 ° C) (Kang and Shapiro 1994), the calcium-binding protein mutant ΔCaBP, and the complemented strain ΔCaBP-C. The protein fractions were separated on a 15% SDS–polyacrylamide gel and analyzed by Western blot (Fig. 3A).

After electrophoresis, proteins were transferred to nitrocellulose membranes and probed with polyclonal anti-GroEL and anti-calmodulin antibodies as previously described. The anti-GroEL antibody detected a protein of approximately 60 kDa in the cytoplasmic fractions of all strains, confirming the integrity of the cytosolic protein preparations (Fig. 3A). The anti-calmodulin antibody recognized a protein of approximately 14.7 kDa in the cytoplasmic fractions of the WT, ΔSecA and complemented ΔCaBP-C strains. The protein analyzed in this study corresponds to CCNA_02838/CC_2752, a predicted multi-EF-hand protein previously identified in *Caulobacter vibrioides*. Therefore, the reactivity observed with anti-calmodulin antibodies is consistent with the presence of conserved EF-hand calcium-binding motifs described for this class of proteins. A band of the similar molecular weight was also detected in the periplasmic fraction of the WT strain. In contrast, no corresponding band was detected in the periplasmic fractions obtained from the ΔCaBP mutant or from the ΔSecA strain grown at 37 ° C (Fig. 3A).

The recognition of this bacterial protein by an antibody raised against eukaryotic calmodulin suggests a structural conservation of epitopes associated with EF-hand motifs, particularly the αE and αF helices that flank the Ca²⁺-binding loop. Importantly, although CaBP was detected in the cytoplasmic fractions of the WT, ΔSecA and complemented ΔCaBP-C strains and in the periplasmic fraction of WT strain, the protein was absent from the periplasmic fraction of both the ΔCaBP mutant and the ΔSecA strain at the restrictive temperature. These results strongly suggest that CaBP is exported to the periplasm through a SecA-dependent secretion pathway, supporting an extracytoplasmic localization of this protein (Fig. 3A). In addition, CaBP exhibited a calcium-dependent electrophoretic mobility shift in SDS–PAGE (Fig. 3B), consistent with Ca²⁺-induced conformational changes, whereas Mg²⁺ or EGTA did not affect its electrophoretic mobility. The observed mobility shift is consistent with calcium-induced conformational changes, a well-documented property of EF-hand calcium-binding proteins.

A bioinformatic survey of EF-hand domain–containing proteins encoded in the *C. vibrioides* genome identified five candidates (Fig. 3C). Among them, CaBP (CCNA_02838; CC_2752) contains three predicted EF-hand domains and a predicted molecular mass of 14.7 kDa, consistent with the band detected in the Western blot assays. Signal peptide prediction using SignalP indicated a signal sequence between residues 1–21, suggesting secretion through the Sec pathway and localization in the periplasmic compartment. Notably, the presence of three EF-hand domains may confer structural similarity to eukaryotic calmodulin-like proteins, which could explain the cross-reactivity observed with the anti-calmodulin antibody used in this study. In contrast, the other EF-hand proteins identified in the genome (CCNA_02234/CC_2151, CCNA_01238/CC_1180, CCNA_02309/CC_2226 and CCNA_02234/CC_2151) contain one or two predicted EF-hand domains and molecular masses ranging from 11.9 to 25.2 kDa, and also present predicted signal peptides according to the bioinformatic analyses, except the probable transmembrane protein CCNA_02234/CC_2192. However, proteomic data reported by Marks et al. (2010) in the *GenBank* displayed these proteins in extracellular fractions, suggesting that they may be secreted or released outside the cell. Together, these analyses indicate that CaBP is the only EF-hand protein predicted to accumulate in the periplasmic space, consistent with the experimental localization results obtained in this study (Fig. 3C).

**Fig. 4:**
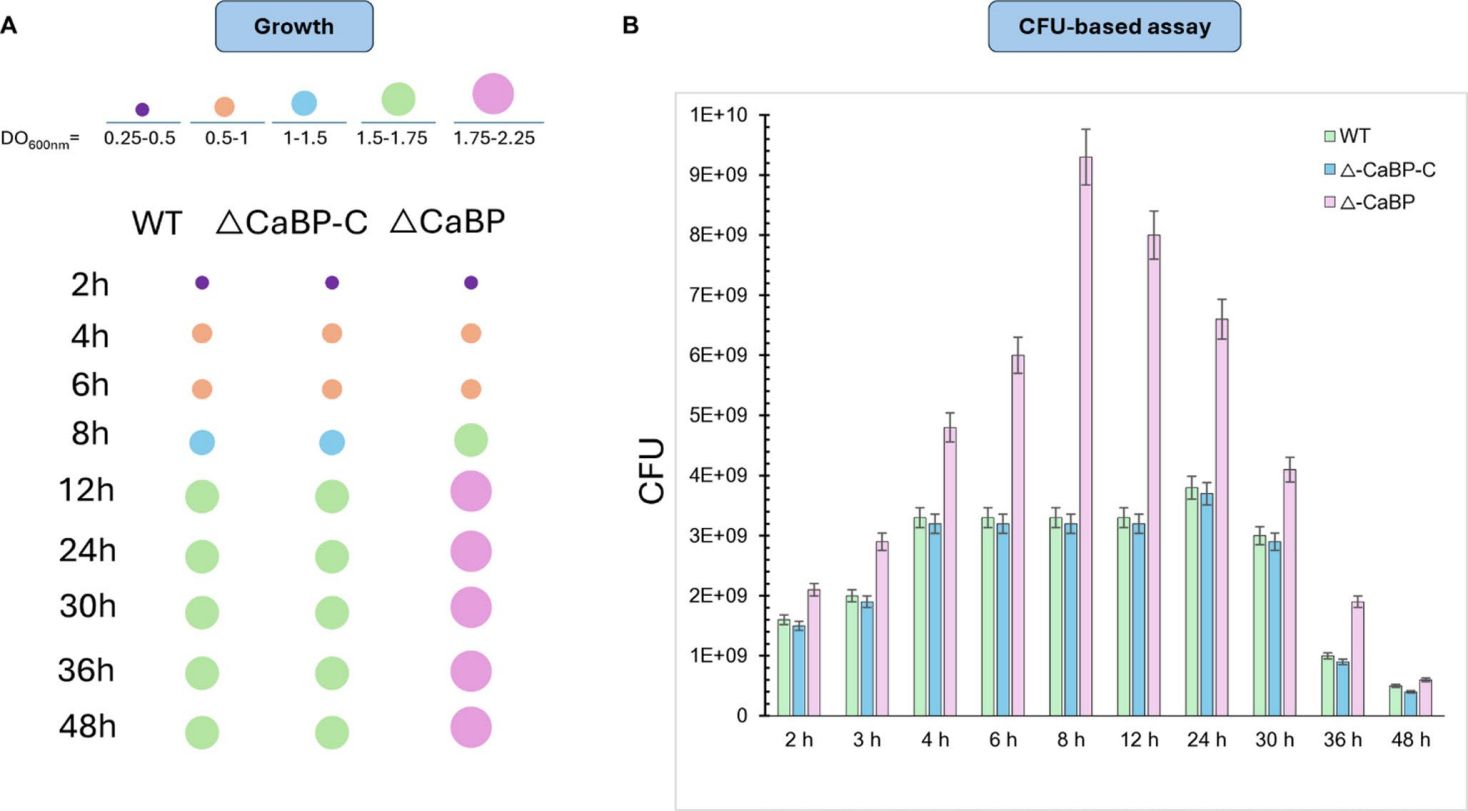
Deletion of the *cabp* gene increases biomass accumulation and enhances cell persistence in *C. vibrioides*. Parental strain (WT), ΔCaBP null mutant, and complemented null mutant(ΔCaBP-C) cells were cultured in M2G medium for 48 h at 30 ° C. At the indicated time points, aliquots were collected to monitor culture growth and cell viability. **A** Growth was monitored by measuring the OD₆₀₀, which provides an estimate of biomass accumulation during the lag, exponential, stationary, and decline phases. **B** Viability assay (CFU-based assay). Aliquots from the cultures shown in (A) were serially diluted (10⁻¹–10⁻⁶) in 0.9% NaCl and plated on PYE agar. After 48 h of incubation at 30 ° C, viable cells were quantified as colony-forming units (CFU), where each colony derives from a single viable bacterial cell present in the original culture. Data represent the mean of three independent experiments performed in duplicate

To evaluate the effect of *cabp* deletion on the growth dynamics of *C. vibrioides*, the parental strain (WT), the ΔCaBP mutant, and the complemented strain (ΔCaBP-C) were cultured in M2G medium and monitored for 48 h (Fig. 4). The deletion of the *cabp* gene was verified by PCR amplification of the deletion junctions and Southern Blot. Importantly, the complemented strain (ΔCaBP-C), in which the wild-type *cabp* gene was reintroduced, restored the wild-type phenotype, confirming that the observed differences are specifically associated with the absence of CaBP. ΔCaBP-C was used as control in all experiments. Bacterial growth was first followed by measuring the optical density at 600 nm (OD₆₀₀), which provides an estimate of biomass accumulation during culture progression (Fig. 4A). All strains exhibited the typical growth phases (lag, exponential, stationary, and decline). However, the ΔCaBP mutant showed a tendency to reach higher OD₆₀₀ values compared to the WT and complemented strains, suggesting increased biomass accumulation in the absence of CaBP.

To determine whether these differences reflected changes in the number of viable cells, aliquots from the same cultures were serially diluted and plated on PYE agar to quantify colony-forming units (CFU) (Fig. 4B). Under these conditions, each colony corresponds to a single viable bacterial cell present in the original culture. The CFU analysis revealed that the ΔCaBP mutant displayed higher numbers of viable cells during the exponential growth phase compared with the WT and complemented strains. At later time points, however, CFU counts declined in all strains as cultures entered the late stationary and decline phases. Together, these results indicate that deletion of *cabp* gene affects biomass accumulation and cell viability during the growth of *C. vibrioides*. Because extracellular matrix production and cell aggregation can influence OD600 measurements, CFU quantification was used as an independent measure of viable cells to support the interpretation of growth differences among strains.

### CaBP and *sigV* gene promoter activity assay

#### CaBP promoter activity under different stresses

**Fig. 5:**
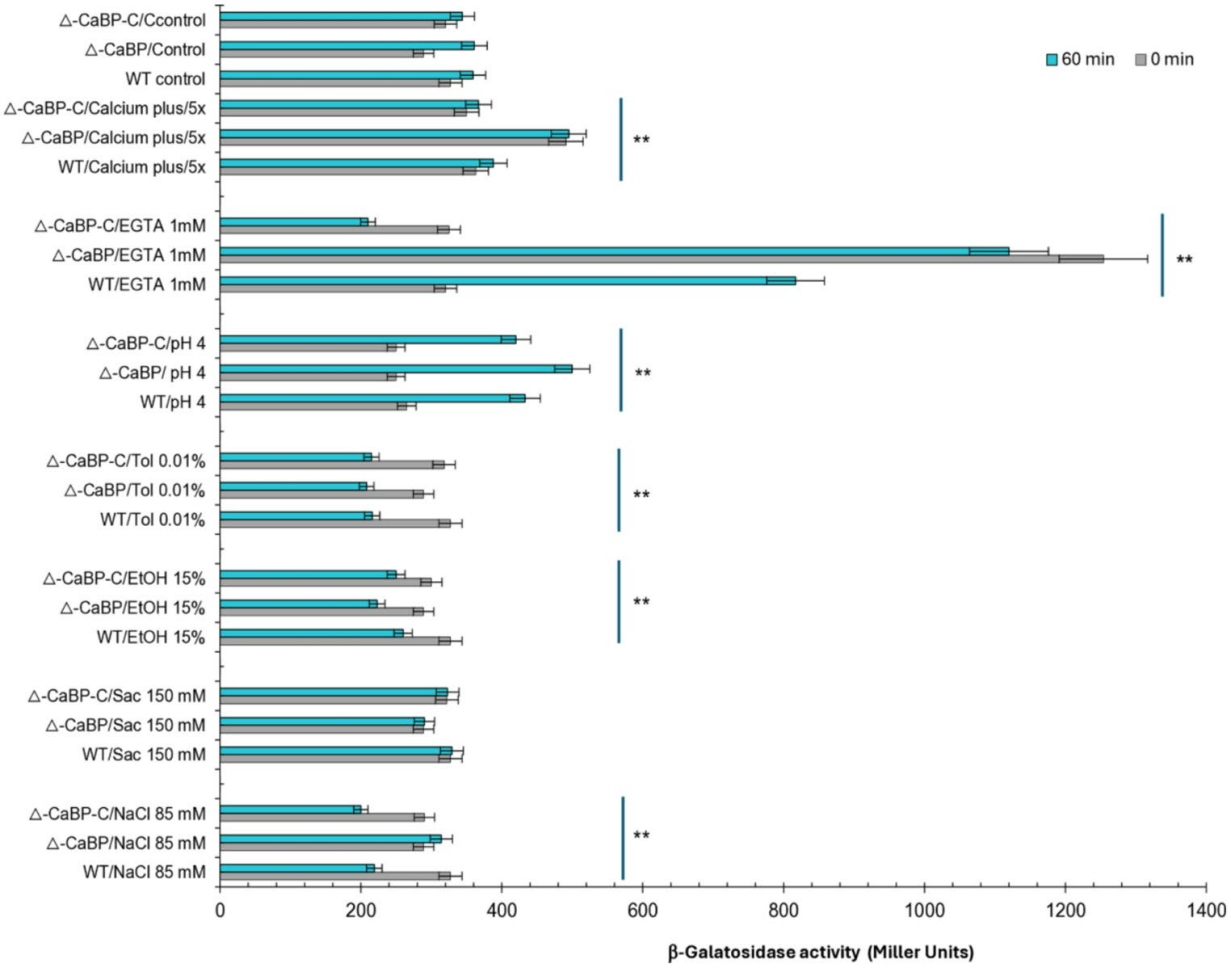
Promoter activity of the *cabp gene* in *C. vibrioides* under stress conditions. β-Galactosidase reporter assays were performed using parental (WT), ΔCaBP, and complemented (ΔCaBP-C) strains. Cells were cultured in M2G medium at 30 ° C to mid-log phase (OD₆₀₀ = 0.4–0.6) and exposed to various stress conditions for 1 h at 30 ° C. Enzymatic activity was quantified using *ο*NPG as the substrate. Data represent mean values from three independent experiments performed in duplicate. Statistical variance was analyzed by one-way ANOVA at a 99% confidence level (*P* < 0.01); asterisks indicate significant differences (*P* < 0.01)

The promoter activity of the *cabp* and *sigV* genes was determined by measuring the *lacZ* reporter gene, which encodes the β-galactosidase enzyme. For this purpose, transcriptional fusions constructed in the *lacZ*290 vector with different promoter regions were used, as previously described. Cells were cultured for 3 h in M2G minimal medium and subjected to treatment with different stressors for 1 h at 30 ° C. Figure 5 shows the β-galactosidase activity (an indicator of promoter activity) in the wild-type, ΔCaBP, and ΔCaBP-C strains. Measurements were made at 0 and 60 min under different stress conditions, including unstressed controls. All strains presented similar basal values ​​(~ 300 units), indicating that the CaBP deletion does not significantly alter basal promoter expression. This reinforces that the differences observed under stress are due to the regulatory response and not to intrinsic differences in expression. CaBP did not participate in the response to osmotic stress caused by NaCl and sucrose. The solvents ethanol and toluene neither induce nor repress CaBP in a CaBP-dependent manner, the response is nonspecific and likely reflects a global decrease in cellular activity under toxicity.

During acid shock stress, there was a significant increase in activity at 60 min in all strains (Fig. 5). The ΔCaBP strain exhibits the highest absolute level, while the WT and complemented strains exhibit similar but lower values. The data suggest that, at acidic pH, CaBP plays a repressive role, attenuating promoter induction. The absence of CaBP releases this repression, resulting in hyperinduction, indicating CaBP-dependent down-modulation under acidity. Like acid stress, in the presence of 1 mM EGTA, and therefore in the absence of calcium via chelator, activity increases strongly in WT and ΔCaBP-C (approximately 1,200 Miller units) but remains significantly lower in ΔCaBP. This pattern persists between 0 and 60 min, suggesting a rapid and sustained response. The promoter is induced by the absence of calcium, and this induction functionally depends on CaBP. Complementation restores the wild-type phenotype. CaBP, therefore, acts as a positive sensor under Ca^2+^ limitation, activating transcription (Fig. 5).

Activity increases in all strains, but WT and ΔCaBP-C exhibit slightly higher values ​​than the mutant. The temporal behavior is stable, indicating a sustained response. In excess Ca^2+^, CaBP also potentiates promoter expression, although the effect is less pronounced than with EGTA. This suggests that the protein responds in a biphasic manner to Ca^2+^ availability, adjusting expression according to the ionic gradient.

### Activity of the *cabp* gene promoter in the presence of EGTA

**Fig. 6:**
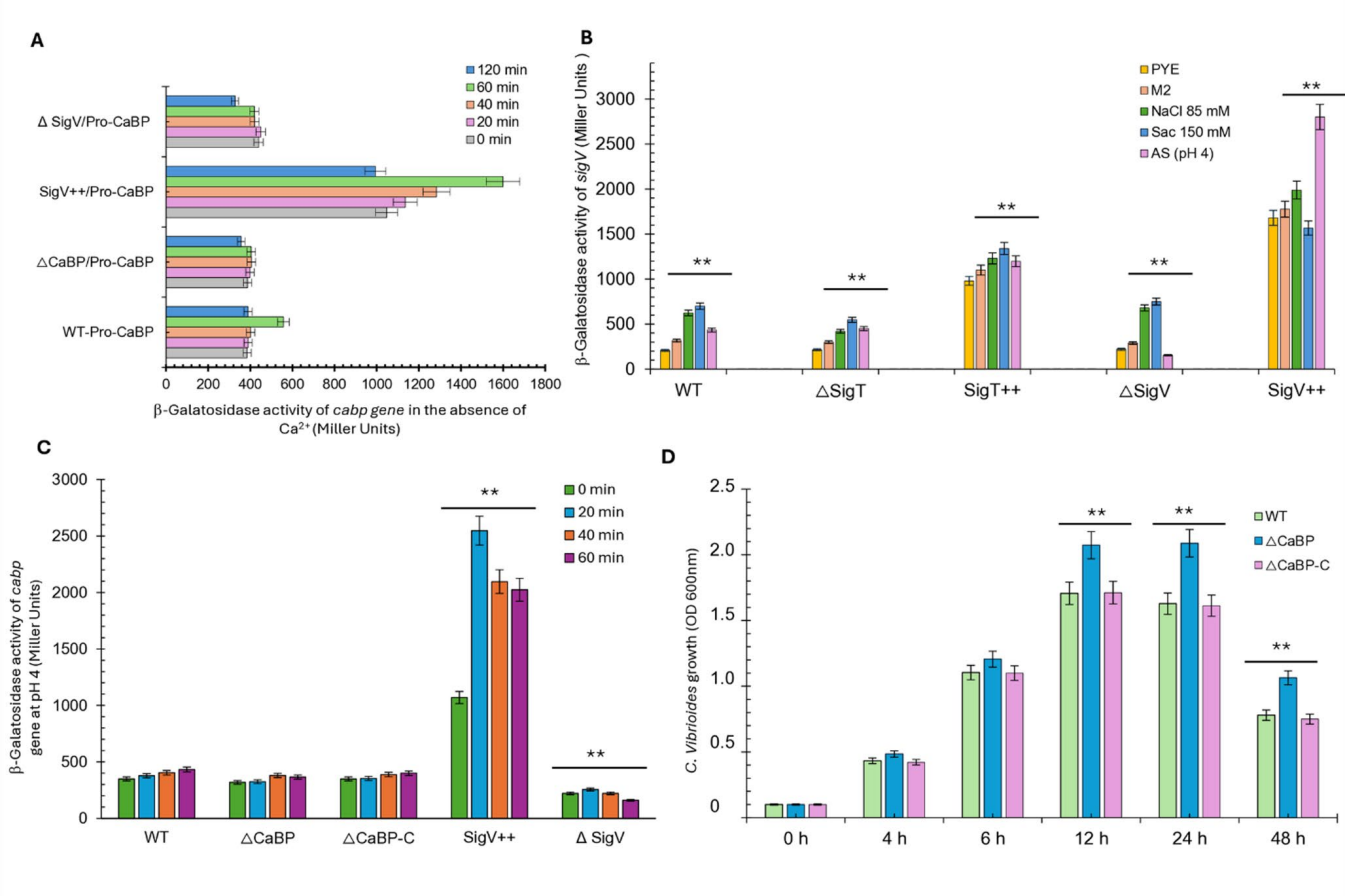
Promoter activity and stress response of *C. vibrioides*. **A** β-Galactosidase assays showing *cabp* and *sigV* promoter activity in wild-type, deletion (ΔSigV, ΔCaBP), and overexpression (SigV++) strains under Ca²⁺ depletion (1 mM EGTA, 120 min). **B ***sigV* promoter activity in WT, ΔSigV, ΔSigT, SigV++, and SigT + + strains exposed to osmotic (85 mM NaCl, 150 mM sucrose) and acid (pH 4) stress for 1 h. **C ***cabp* promoter activity after acid shock (pH 4, 0–60 min). **D** Growth recovery after 1 h acid stress, monitored by OD₆₀₀ nm for 48 h. Data represent mean ± SD (*n* ≥ 3); ANOVA, *P* < 0.01; asterisks denote significant differences

The *cabp* gene transcriptional fusion was introduced into the WT, ΔCaBP, SigV++ (overexpressing *sigV*), and ΔSigV strains, incubated in M2G minimal medium with 1 mM EGTA. The WT and ΔCaBP strains presented similar profiles, indicating that CaBP does not directly regulate its own promoter in the absence of calcium (Fig. 6A). The SigV + + strain exhibited strong induction of promoter activity, peaking at 60 min (~ 3-fold the WT level), while ΔSigV maintained low and stable values. These results indicate that protein SigV is the main Sigma-factor activating *cabp* transcription. The transient peak of expression at 60 min followed by a decline is typical of responses mediated by inducible Sigma-factors, suggesting negative autoregulation or exhaustion of the stimulus.

### *SigV* promoter activity in different stresses

Considering the regulatory relationship between the *cabp* gene and the SigV factor, defining the stresses that induce promoter activity may contribute to understanding how *cabp* can be regulated. Therefore, the transcriptional fusion containing the *sigV* gene promoter was evaluated in different strains: the wild-type (WT) and mutant strains overexpressing the *sigV* and *sigT* genes. The analysis of *sigT* strains is since this sigma factor has already been characterized as the master sigma factor in the stress response among ECF factors and on the evidence that both the *cabp* and *sigV* genes have promoter regions with a conserved consensus for SigmaT binding (Fig. 2). The WT, ΔSigV, SigV++, ΔSigT, and SigT + + strains were compared for 1 h under different conditions: PYE, M2, 85 mM NaCl, 150 mM Sucrose, and acid stress at pH 4. The measured β-Galactosidase activity reflects the strength of the *sigV* promoter under each condition (Fig. 6B). The wild-type strain showed moderate basal levels of activity, slightly higher in minimal medium and under osmotic stress (NaCl/Sucrose), suggesting that the *sigV* promoter responds discreetly to osmotic and nutritional stresses (Fig. 6B). Overexpression of *sigT* promotes clear induction of *sigV* promoter activity under all conditions tested, approximately 2- to 3-fold greater than that observed in the wild-type, with a peak under acid shock stress. This indicates that protein SigT acts as a positive transcriptional activator of *sigV*, possibly regulating its expression under acid and osmotic stress. Furthermore, *sigV* deletion strongly reduces promoter activity in all media, with no induction under stress. This suggests that *sigV* exhibits positive autoregulation, dependent on its own presence. Overexpression of *sigV* causes a massive increase in promoter activity, particularly at pH 4, where levels reach approximately 5–6 times the WT value (Fig. 6B). This result strongly suggests that the *sigV* transcription factor is the primary Sigma factor activating its own transcription, characterizing an acid-stress-inducible positive feedback loop.

Because gene expression in *Caulobacter* is tightly coordinated with the cell cycle, future studies using synchronized populations may help determine whether the CaBP–SigV regulatory circuit exhibits stage-specific modulation. However, stage-resolved regulation was beyond the scope of the present study.

### Cabp-promoter activity in response to acid shock (pH 4)

The involvement of both the *cabp* and *sigV* genes in the acid shock response was confirmed. The transcriptional fusion containing the *cabp* gene promoter was inserted into wild-type strains, *sigV* and *cabp* deletion mutants (ΔSigV; ΔCaBP), *sigV* overexpression mutants, and the ΔCaBP complemented mutant. The strains were grown to log phase and subjected to 60 min of acid shock. The activity of the *cabp* gene promoter was assessed at 20-min intervals. The *cabp* gene promoter activity increases subtly and gradually over 60 min at acidic pH, indicating that acid stress activates the *cabp* promoter. Individual deletion in the *cabp* mutant did not abolish promoter induction, suggesting functional redundancy or that this protein does not strongly regulate the promoter under acid stress. However, when *sigV* gene is overexpressed, a much more intense and rapid activation occurs, reaching peaks of ~ 2,500 in 20 min, confirming that *sigV* factor strongly modulates the *cabp* promoter (Fig. 6C). Depletion of the *sigV* gene, on the other hand, drastically reduces induction by acidic pH, reaching only 132 U Miller after 60 min. The data suggest that *cabp* promoter activity is *sigV*-dependent under acid stress. The calcium-binding protein promoter is inducible by acidic pH, and SigV is the main positive regulator, while CaBP has minimal effect (Fig. 6C).

Acid-shocked cells were incubated in M2G medium and growth was monitored for up to 48 h under normal physiological conditions (Fig. 6D). The ΔCaBP mutant grows faster and larger than WT and ΔCaBP-C, reaching OD ~ 2.1, suggesting that the absence of CaBP may allow adaptation or compensatory tolerance to acid shock. The complemented ΔCaBP mutant strain showed similar growth to the WT. The parental strain grows similarly to the complemented mutant, reaching OD ~ 1.7, suggesting that the presence of CaBP modulates adaptation to acid shock. The increased *cabp* promoter activity in WT and the deletion mutant suggests that gene induction by acid stress primes cells for adaptation. Interestingly, ΔCaBP reaches a higher biomass than WT, suggesting that the absence of CaBP may trigger compensatory mechanisms for survival to acid shock (Fig. 6D).

### Evaluation of cabp and *sigV* gene expression by qRT-PCR

**Fig. 7:**
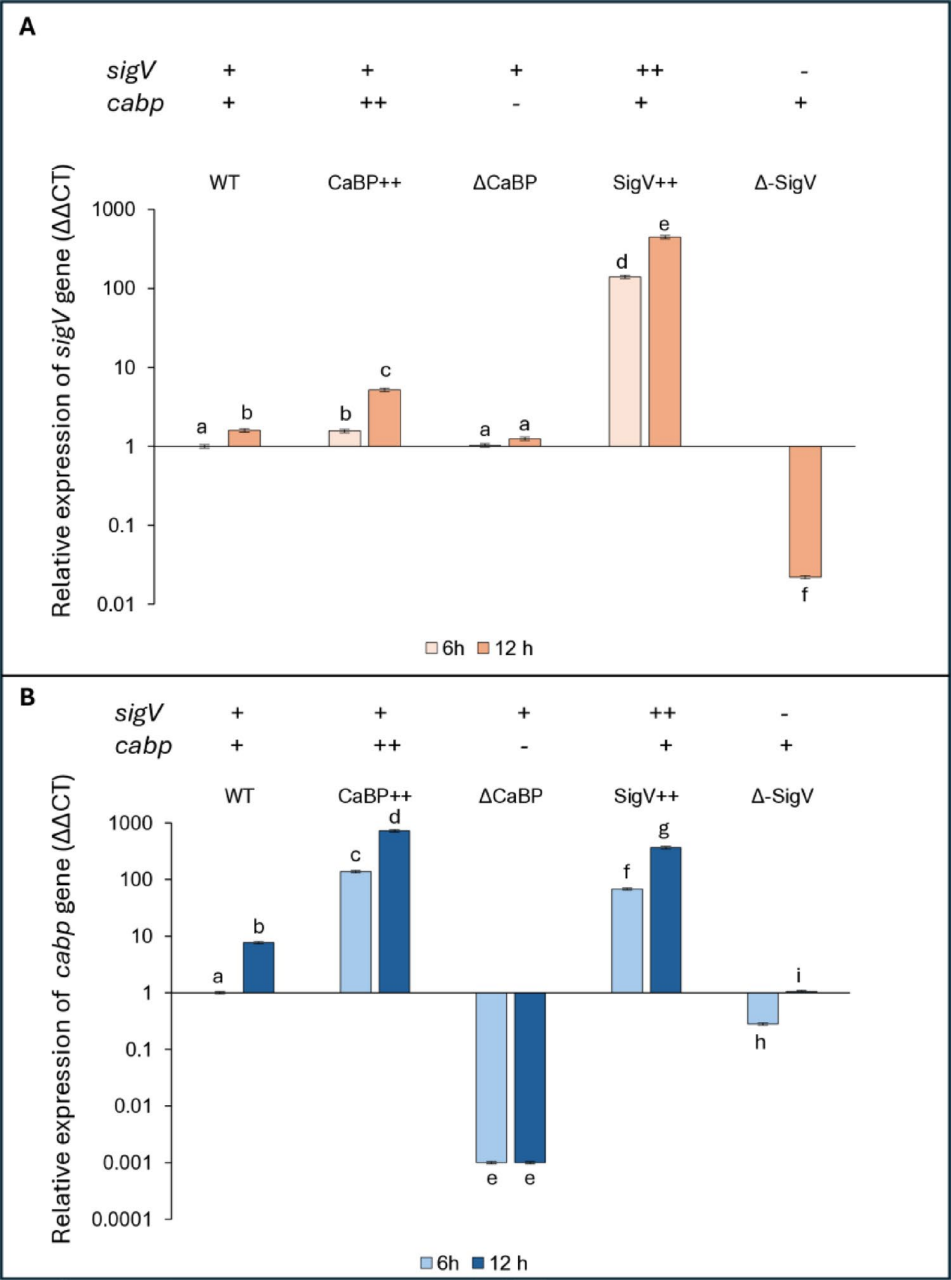
Quantitative expression of *sigV* and *cabp* in *C. vibrioides* parental and mutant strains. Relative transcript levels were quantified by RT-qPCR using 16 S rRNA as the internal control. Values are shown on a semi-logarithmic scale and represent the mean of three independent experiments, each analyzed in duplicate. Cultures were grown in M2G medium. Statistical significance was assessed by one-way ANOVA at a 99% confidence level (*P* < 0.01); identical letters indicate non-significant differences, while different letters denote significant variation. Symbols (–), (+), and (++) indicate absence, basal, and elevated gene product levels, respectively

Parental (WT) cells and *cabp* and *sigV* deletion and overexpression mutants were grown for 24 h, and samples were collected at 6 and 12 h for total RNA extraction, followed by qPCR assays using specific primers. Quantitative analysis of gene expression obtained by qPCR reveals a clear regulatory pattern involving *sigV* and *cabp*, which behave as interdependent components of a reciprocal activation circuit (Fig. 7A-B). For *sigV*, the wild-type strain shows basal expression at 6 h, while CaBP overexpression leads to a slight increase, suggesting that this protein may act as an initial modulating signal for *sigV* gene induction. The absence of CaBP does not significantly alter expression at this timepoint, indicating that the gene is not essential for the initial activation of *sigV*. In contrast, the *sigV*-overexpressing strain exhibits an extremely high increase in expression, demonstrating a strong autoregulatory mechanism, while *sigV* deletion results in a complete absence of transcripts, validating the experimental control (Fig. 7A). After 12 h, *sigV* expression increases in the wild-type strain, indicating temporal induction. This increase is more pronounced in CaBP++, suggesting that CaBP positively affects *sigV*, possibly by amplifying the stress signal that activates the SigmaV factor. The absence of CaBP leads to a slight reduction in expression, and *sigV* overexpression maintains very high levels, confirming *sigV*’s ability to autoinduce itself (Fig. 7A).

.

For *cabp*, the pattern is similar but inverted in functional hierarchy. At 6 h, the wild-type strain exhibits basal expression, while CaBP++ (pAS22 + *cabp*) shows strong overexpression, and gene deletion results in almost no expression. Overexpression of *sigV* significantly increases *cabp* expression, and deletion of *sigV* causes a marked reduction, indicating that SigV factor positively affects expression *cabp.* Within 12 h, physiological induction occurs in WT, and expression becomes extremely high in CaBP + + and ΔSigV + + strains, while the absence of SigV protein restores near-baseline levels, suggesting that SigV factor is decisive in the early phase of the response, but there may be partial compensation later (Fig. 7B).

In summary, the results indicate a positive regulatory loop between SigV and CaBP: *sigV* strongly induces *cabp*, while CaBP potentiates *sigV* expression. Both also exhibit self-promotion, configuring a feedback loop that can amplify the response to stress signals. Deletion of *sigV* gene leads to reduction of *cabp*, and overexpression of CaBP stimulates *sigV*, characterizing a cooperative relationship that is likely associated with the adaptive response and maintenance of cellular homeostasis under stress conditions (Fig. 7A and B).

### Characterization of the mutant by microscopy

**Fig. 8:**
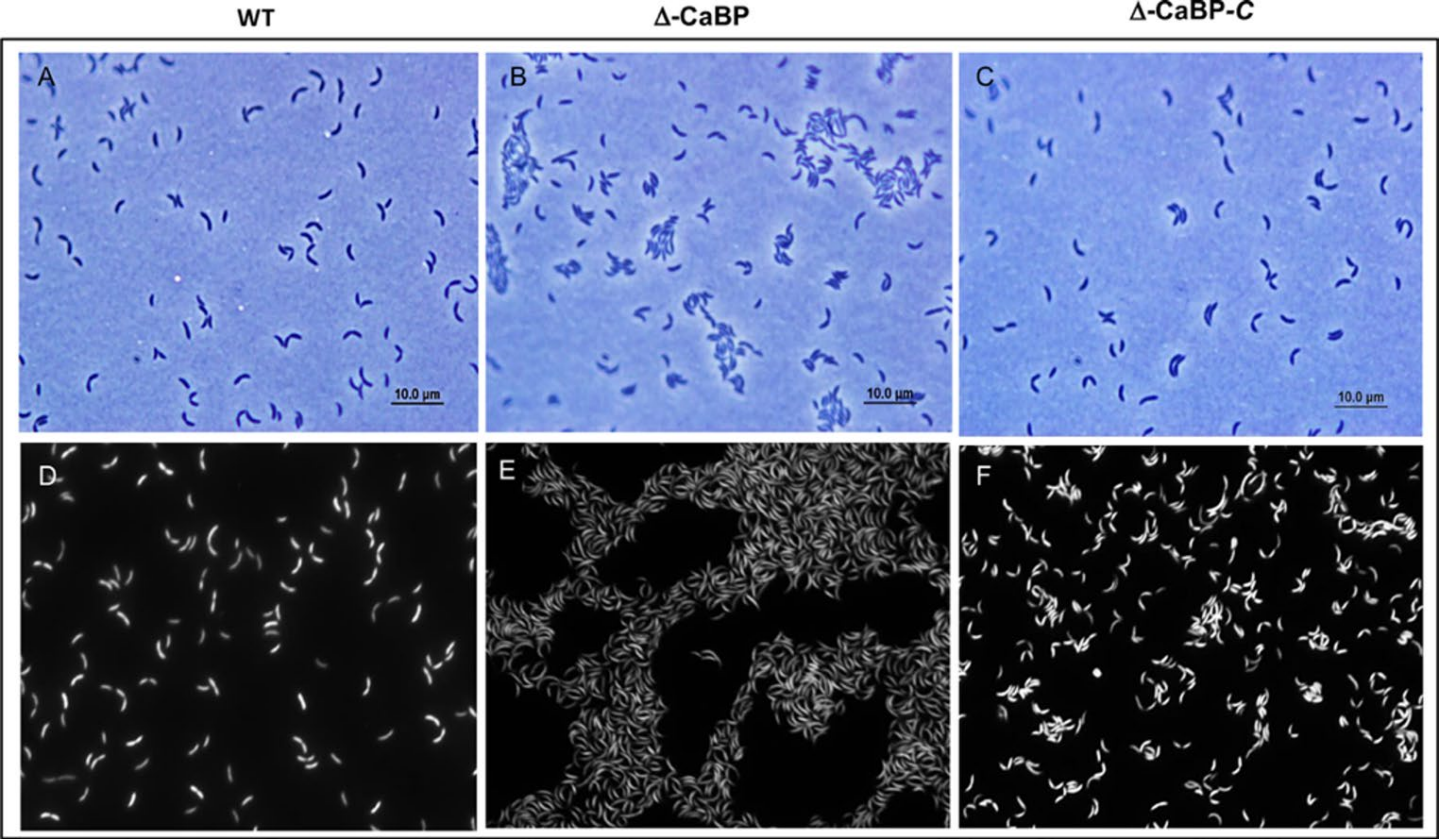
Fluorescence microscopy of *C. vibrioides* strains. Microscopic analysis of the parental strain NA1000 (WT), ΔCaBP mutant, and complemented strain ΔCaBP-C was performed at early stationary phase (OD₆₀₀ = 1) in M2G minimal medium. Cells were stained with FM 595 (1 µg mL⁻¹), a lipophilic dye that labels the cytoplasmic membrane. The upper panels display phase-contrast images, while the lower panels show fluorescence images acquired under excitation at 515–560 nm

The findings show regular bacterial cell membrane morphology for the wild-type bacterial strains WT, ΔCaBP, and ΔCaBP-C (Figs. 8A-F). Very subtly, possible alterations in bacterial cell morphology were observed in the mutant strain for the CaBP protein (ΔCaBP). Unlike the wild-type strain and the complemented mutant, which present well-defined cell morphologies, especially regarding the characteristics of pre-divisional cells, in the panel showing the cluster of cells mutant for the *cabp* gene (Fig. 8E), the cells appear to be slightly more curved, as if the two ends of a stalk or predivisional cell were anchored to a substrate. In this sense, it has been hypothesized that CaBP protein depletion may play a role in bacterial biology as well as in a set of events involved in cell differentiation and division.

In short, a very large and complex series of events controlling cell division and differentiation that has been studied for decades by different research groups using *C. vibroides* as a model organism. The complemented mutant strain shows clear evidence of functional complementation, with no apparent differences between the parental strain and the complemented mutant (Fig. 8). Morphometric analysis of the cells did not reveal significant differences in the sizes of the mutant cells compared to the wild-type cells. Because this result is very subtle and apparently inconclusive regarding how the depletion of CaBP may have affected bacterial morphology, and because the events related to the biology of cell growth and differentiation in *C. vibrioides* involve a vast set of well-characterized genes, an additional microscopic approach was used in this study to understand the persistence of the mutant strain and its apparent greater growth ability compared to the wild-type strain (Figs. 4 and 6D).

**Fig. 9:**
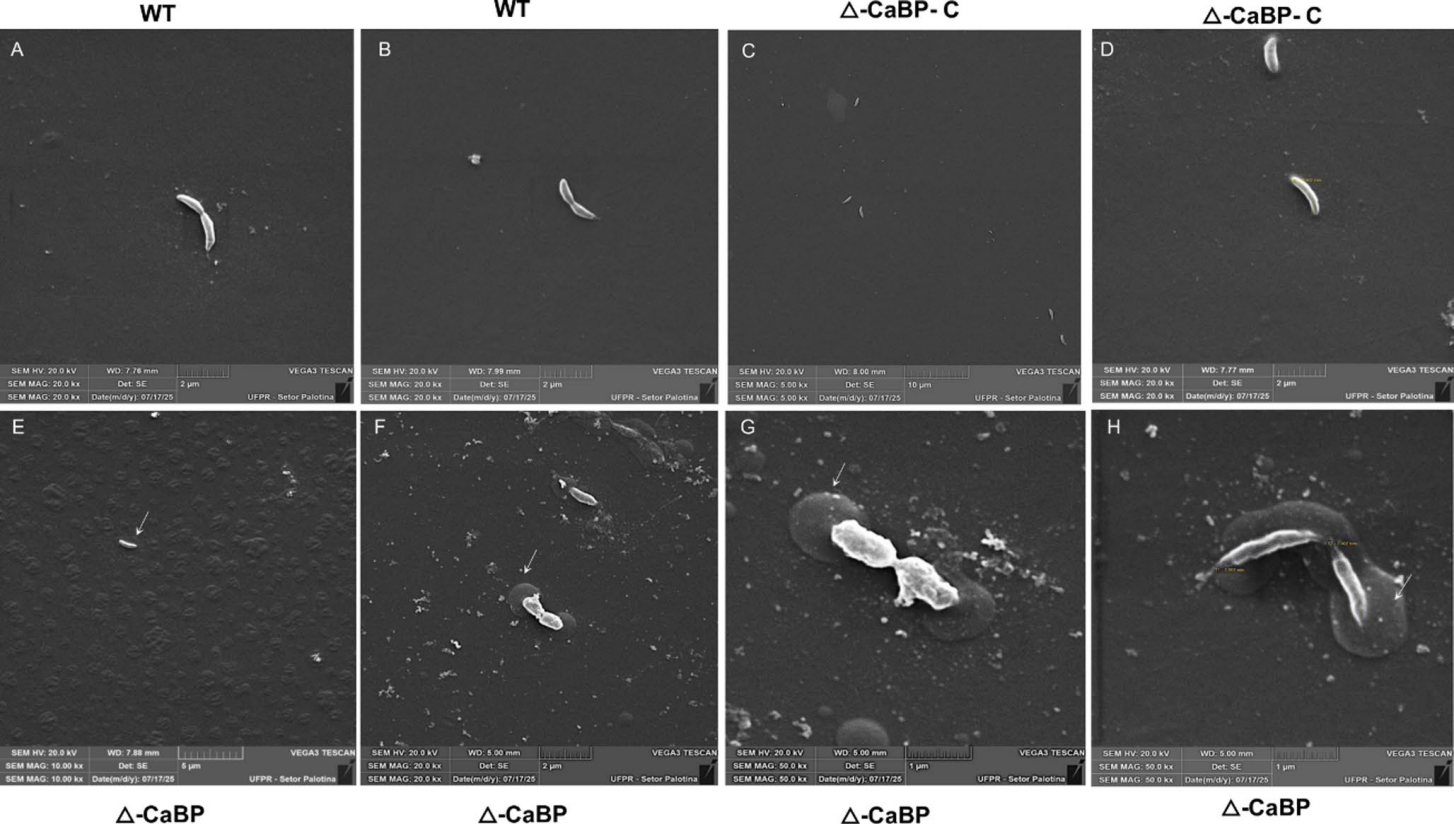
Scanning electron microscopy (SEM) of *C. vibrioides* strains reveals altered adhesion and polarity. Bacterial strains (WT, ΔCaBP, and ΔCaBP-C) were cultivated on tissue culture plates in PYE medium. Adherent cells were fixed and processed for SEM as described in the Material and Methods section. After dehydration, plate fragments were sectioned and imaged at different magnifications. **A–B** Wild-type cells at 20,000× magnification showing normal morphology and polar adhesion. **C-D** Complemented ΔCaBP-C strain displaying restoration of the wild-type phenotype. **E** ΔCaBP mutant at 5,000× magnification showing a cell attached at both poles and forming a “mountain-like” structure, indicative of excessive holdfast secretion. **F**–**G** The same cell imaged at 20,000× and 50,000× magnification, respectively, confirming bipolar adhesion. **H** Additional ΔCaBP cells at 50,000× magnification showing clear loss of symmetry and adhesion at both poles

Scanning Electron Microscopy (SEM) assays were conducted using *C. vibroides* cells previously synchronized using tissue culture plates, low agitation, and transfer of non-adherent motile cells to the test plates (Fig. 9). The motile cells obtained by synchronization were inoculated from PYE medium into mini tissue culture plates. The cells were diluted to an optical density of 0.1 and incubated in 1 mL of PYE medium for 6–8 h under gentle shaking at 20 rpm. After this period, the culture media were discarded, washed, and fixed according to the methodology developed for this SEM analysis and described in methods. The tissue culture plates were cut into 0.5 × 0.5 cm wide squares using a sterile scalpel previously heated in a hot flame and treated for scanning electron microscopy. The objective of the analysis was to emphasize the observation of pre-divisional cells in the tissue culture plates and to verify possible more direct variations in the morphology of the ΔCaBP strain compared to the parental strain.

Cells of the parental strain show typical morphology and characteristic size at 20,000x magnification (Fig. 9A-C). The complemented mutant, as confirmed in all analyses in this work, shows direct similarity to the parental strain, confirming the reversion of the original phenotype (Fig. 9C-D). The parametric data for wild-type and mutant cells were constant at all observed magnifications, i.e., a length of 0.002 mm (2 µM). Interestingly, SEM analysis showed that the tissue culture plates previously occupied by the ΔCaBP mutant strain presented a generalized alteration indicating an overproduction of adhesive compounds commonly secreted by *C. vibrioides* (Fig. 9E) for cell stalk attachment. It is important to emphasize that the methodological approach developed for SEM analysis ensures that the cells are in the form in which they originally grew and adhered.

**Fig. 10:**
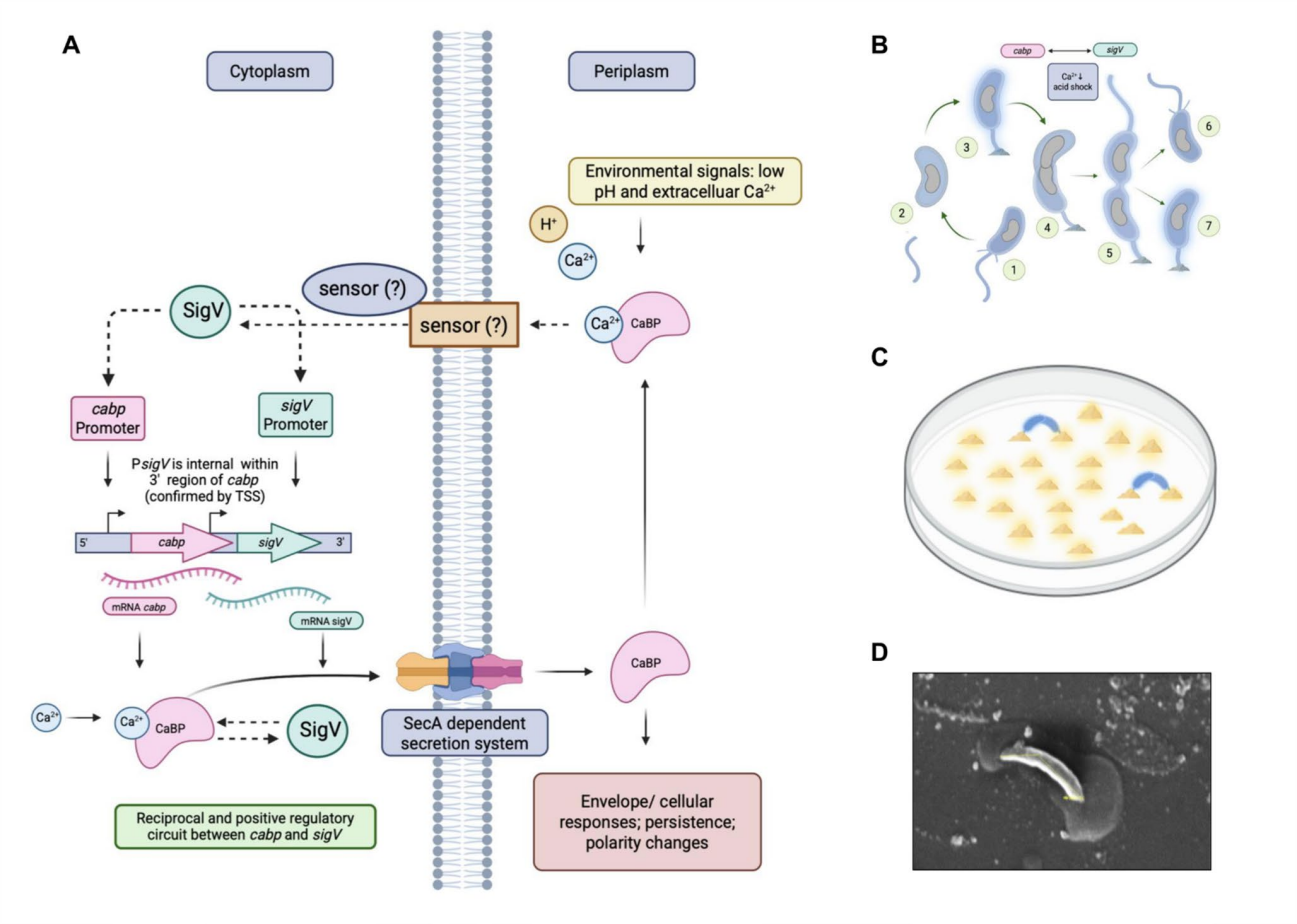
Hypothetical model for the CaBP-SigV regulatory circuit in *C. vibrioides*. Solid arrows display mechanism experimental demonstrated in the model and dashed arrows show events supported by physiological/genetic data. **A** Environmental stresses such as calcium limitation and acid stress activate the extracytoplasmic function sigma factor SigV. SigV positively modulates *cabp* transcription, leading to increased production of the periplasmic Ca²⁺-binding protein CaBP. CaBP may sense calcium availability in the periplasm and modulate envelope stress signaling. Because CaBP is predicted to be periplasmic, the regulatory effect on *sigV* transcription is likely mediated indirectly through an envelope-associated signaling pathway involving an unidentified membrane component (?), possibly an anti-sigma factor typical of ECF systems. This creates a Ca²⁺- and pH-responsive regulatory loop integrating environmental signals into stress adaptation. **B** The *C. vibrioides* life cycle involves asymmetric division, producing morphologically distinct cells. Motile swarmer cells (1) possess fimbriae and a polar flagellum for movement in aquatic environments. Under nutrient-rich conditions, the cell retracts its fimbriae, loses the flagellum (2), and develops a stalk at the same pole (3), becoming sessile. This cell initiates DNA replication and elongation (4), generating a predivisional cell (5) that divides asymmetrically to yield a new motile swarmer (6) and a stalked mother cell (7). **C** The model also depicts that CaBP depletion disrupts polarity, leading to double-stalk formation and aberrant secretion of material consistent with adhesive matrix at both poles. **D** This phenotype manifests macroscopically as dense clusters or “mountain-like” biofilm formations on culture surfaces

The cells were not collected and placed on a support for SEM analysis; they grew in the culture plate and were fixed and analyzed on these plates by SEM. In addition to the observation of the “mountain” effect (Fig. 10B) seen in the plates containing the ΔCaBP mutant strain (Figs. 9F-H and 10B-D), the microscopy performed in which the cells were able to remain fixed suggests a bipolar adhesion by the pre-divisional cell (Fig. 9F-H), which was not seen in the pre-divisional cells of the wild-type and ΔCaBP-C strains (Figs. 9A-E). The ΔCaBP strain produced a substantially larger amount of extracellular material; however, cells were not observed attached to the plate via their stalk. Although increased extracellular material was observed, no direct adhesion or biofilm assays were performed in this study; therefore, no functional conclusions regarding adhesion are drawn.

Based on the genetic, transcriptional, and physiological evidence obtained in this study, a regulatory model integrating CaBP and SigV is proposed (Fig. 10). In this model, SigV activates *cabp* transcription, while CaBP enhances *sigV* expression, establishing a regulatory feedback loop responsive to calcium limitation and acid stress. Because CaBP is localized in the periplasm whereas sigma factors function in the cytoplasm, signal transmission likely involves an intermediary membrane-associated component linking periplasmic calcium sensing to SigV-dependent transcriptional regulation.

## Discussion

### Structure and conservation of CaBP

Although our data demonstrate a clear functional relationship between SigV and *cabp* expression, we did not directly assess DNA binding of SigV to the *cabp* promoter. Therefore, we cannot exclude the possibility that this regulation occurs indirectly through additional regulatory components. Classical genetic approaches have long established that regulatory relationships can be robustly inferred from transcriptional and genetic evidence, even in the absence of direct DNA-binding assays. In this study, we describe a physiological regulatory interaction between SigV and *cabp* under stress conditions.

This study highlights how non-canonical genomic architectures can underlie independent gene regulation and may be overlooked without direct experimental validation. Consistent with our findings, transcriptomic analyses by Bharmal et al. (2020) mapped the *sigV* transcription start site within the coding sequence of *cabp*, providing independent evidence that the *sigV* promoter is embedded within *cabp* and supports independent transcription despite gene overlap.

Here, we identified and characterized a previously unstudied calcium-binding protein in *C. vibrioides*, designated CaBP, revealing its involvement in growth regulation and adaptive response to environmental stresses. In silico analyses showed that CaBP contains three EF-hand motifs, two of which retain the canonical Ca²⁺-coordinating residues, while the central motif presents a non-conservative substitution (Arg⁶), possibly responsible for differentiated affinities between the domains. The presence of a Sec/SPI-type signal peptide indicates a likely periplasmic or extracellular localization, which was experimentally confirmed by the absence of the protein in the periplasmic extracts of the ∆CaBP and ∆SecA mutants (Kang and Shapiro 1994). The recognition of CaBP by a eukaryotic anti-calmodulin antibody (Simão and Gomes 2001) in the periplasmic protein extract reinforces the structural conservation of the EF-hand domains, suggesting that Ca²⁺-binding motifs of bacterial origin share conformational epitopes with eukaryotic regulatory proteins.

Furthermore, the protein investigated in this study corresponds to CCNA_02838/CC_2752, which has been previously classified as a multi-EF-hand protein in *Caulobacter vibrioides* through sequence-based analyses (Michiels et al. 2002). Such proteins have been proposed as bacterial calmodulin-like proteins and are thought to participate in calcium-dependent regulatory processes, including responses to environmental stress. Therefore, our objective was not to establish immunological specificity using a CaBP-specific antibody, but rather to functionally characterize a member of this class of calcium-binding proteins. In this context, the use of anti-calmodulin antibodies is consistent with previous studies, where calmodulin-like proteins in bacteria were initially identified based on immunological cross-reactivity.

Moreover, our conclusions do not rely solely on antibody-based detection but are supported by multiple independent lines of evidence, including sequence homology, calcium responsiveness, and physiological phenotypes. Therefore, the observed antibody reactivity reflects conserved structural features of EF-hand proteins rather than nonspecific detection.

Importantly, the interpretation of calcium binding in this study does not rely solely on the electrophoretic mobility shift. Instead, it is supported by multiple independent observations, including sequence-based identification of EF-hand motifs, calcium-responsive behavior, and associated physiological phenotypes. Together, these findings provide a consistent framework supporting the role of this protein as a calcium-binding factor.

The calcium-dependent mobility shift observed in this study provides qualitative evidence of calcium-induced conformational changes, which are a hallmark of EF-hand proteins. We acknowledge that this approach does not provide quantitative binding parameters such as affinity constants. However, our objective was not to perform a detailed biophysical characterization of calcium binding, but rather to assess the functional relevance of this protein in a physiological context.

Although the bioinformatic analysis identified additional EF-hand–containing proteins in *C. vibrioides*, only the 14.7 kDa protein encoded by the *cabp* gene displayed the characteristic biochemical properties expected for a calcium-binding regulatory protein, a Calmodulin-like protein. In particular, the protein exhibited a clear calcium-dependent mobility shift in SDS-PAGE, a feature commonly observed in EF-hand proteins undergoing conformational changes upon Ca²⁺ binding (Simão and Gomes 2001). Moreover, this protein was specifically recognized by an anti-Calmodulin antibody, whereas other predicted EF-hand proteins from *C. vibrioides* were not detected under the same conditions. This selective recognition suggests that, despite the presence of multiple EF-hand proteins in the genome, CaBP shares structural or conformational epitopes with calmodulin that are not conserved in the other family members (Simão and Gomes 2001). Together, these observations reinforce the notion that CaBP represents a distinct EF-hand calcium-binding protein in *C. vibrioides*, combining bioinformatic features with biochemical behavior consistent with calcium-dependent conformational regulation. The Ca²⁺-dependent mobility shift observed in SDS–PAGE provides experimental evidence supporting calcium binding by CaBP. However, detailed biophysical characterization will be required to determine the affinity and stoichiometry of Ca²⁺ coordination by the predicted EF-hand motifs.

### Physiological role and parallels with bacterial Ca²⁺ sensors

Depletion of the CaBP resulted in accelerated growth, increased maximum biomass, and greater persistence in the stationary and declining phases, while genetic complementation restored the wild-type phenotype. These findings indicate that CaBP acts as a negative modulator of metabolism and cellular physiological balance. Recent evidence indicates that bacterial EF-hand proteins can act as transcriptional sensors that adjust metabolism according to Ca²⁺ availability, as observed for EfhP in *Pseudomonas aeruginosa* (Burch-Konda et al. 2024). Thus, it is plausible that the CaBP of C. *vibrioides* plays an analogous role, integrating ionic signals into regulatory mechanisms of growth and survival.

However, an important limitation of the present study is that the molecular link connecting CaBP to the SigV regulatory pathway remains unresolved. In the model proposed in Fig. 10, this connection is represented by a putative sensor component (a transmembrane protein indicated by a question mark) which could correspond to a typical anti-sigma factor or a related regulatory element. Anti-sigma systems are widely employed in bacteria to modulate the activity of extracytoplasmic function (ECF) sigma factors in response to environmental signals (Mascher 2025). Although our data support the idea that CaBP influences cellular physiology in a calcium-dependent manner, the mechanism by which this signal would be transmitted to SigV remains to be determined. Therefore, the model presented here should be considered a working hypothesis that integrates our experimental observations with established regulatory paradigms, while future studies will be required to identify the molecular components that mediate this connection.

### Promoter activity and biphasic behavior of CaBP

Transcriptional fusion assays revealed that the *cabp* promoter is induced by both calcium depletion (1 mM EGTA) and acid stress, indicating that the gene responds to multiple environmental stimuli. Promoter activation in the absence of calcium depends on the functional presence of CaBP itself, suggesting positive regulatory feedback, while under acidic pH, CaBP plays a repressive role, attenuating transcription induction. Thus, the protein appears to act in a biphasic manner, either activating or repressing transcription, depending on Ca²⁺ availability and the type of cellular stress. The dual responsiveness of the *cabp* promoter to calcium depletion and acid stress is particularly significant, as both conditions can perturb periplasmic ionic homeostasis and envelope stability. This regulatory duality is consistent with the model proposed by Agaras and collaborators (2023), who describe bacterial CaBPs as multifunctional sensors capable of modulating specific and broad-spectrum responses to environmental variations. These observations suggest that CaBP-dependent regulation may operate within a broader transcriptional network associated with envelope stress responses, prompting us to examine the regulatory relationship between CaBP and the ECF sigma factors SigV and SigT.

### Regulatory interdependence between cabp, sigV and sig^T^

The results of gene expression and β-galactosidase assays demonstrated a clear interdependence between CaBP and the extracytoplasmic sigma factor SigV. The SigV factor influences *cabp* transcription, while CaBP overexpression potentiates *sigV* gene expression, configuring a positive feedback loop. Both genes show evidence of autoregulation, constituting a regulatory module that integrates ionic (Ca²⁺) and environmental (pH) signals into the bacterial envelope response. This type of coupling between sensor proteins and ECF sigma factors was extensively discussed by Mascher (Mascher 2025) who highlighted the ability of these systems to establish amplification loops under stress conditions, conferring a rapid and self-reinforcing response to the cell. Furthermore, the activation of *sigV* gene by SigT under multiple stresses indicates a functional hierarchy among sigma factors of the ECF family, in which SigT acts as a master regulator and SigV as a specific mediator of the acid response and calcium homeostasis, in accordance with the evolutionary model of functional diversification of ECFs proposed recently (Mascher 2025).

Previous studies have demonstrated that the SigT coordinates a regulatory cascade involving multiple ECF sigma factors in *C. vibrioides*, including SigU, whose promoter contains a characteristic SigT-binding motif (Alvarez-Martinez et al. 2006, 2007; Lourenço et al. 2011). These promoters share conserved − 35 and − 10 elements, allowing cross-recognition and the establishment of hierarchical regulatory loops among ECF sigma factors. Such architecture enables SigT to function as a master regulator of extracytoplasmic stress responses, initially activating downstream sigma factors that subsequently reinforce their own expression through positive autoregulatory feedback (Mascher 2025).

In the present study, we demonstrated that both *sigV* and *cabp* promoters contain motifs consistent with the *sigU* consensus, and their expression is dependent on SigT. In fact, the − 35 region of the *sigV* transcription factor differs by only one nucleotide from the sequence described by Alvarez-Martinez (Alvarez-Martinez et al. 2007). Furthermore, the comparison of these promoters expands the consensus in the − 35 region for ECF-type sigma factors, as they present four more identical nucleotides, ttcg (consensus: GGAAG/CCttcg) (Fig. 2C). This may direct new mutagenesis approaches to better understand the recognition of the *sigV* gene promoter by SigV itself or SigT. Furthermore, these data strongly indicate that *cabp* and *sigV* also integrate the SigT-controlled regulon and that this would indeed be the majority sigma among ECFs in *C. vibrioides.* Although the genetic data strongly support positive regulation of by SigV, direct binding of SigV to the *cabp* promoter has not yet been experimentally demonstrated. Likewise, the functional requirement of the predicted − 35/−10 promoter elements remains to be validated by promoter mutagenesis and DNA-binding assays such as EMSA, footprinting, or ChIP-based approaches.

### Morphological changes and implications for the cell envelope

Morphological and scanning electron microscopy analyses revealed that the absence of CaBP causes subtle but consistent changes in cell asymmetry and the secretion of adhesive polymers. The ∆CaBP mutant showed excessive accumulation of extracellular material and partial loss of the polarity typical of pre-divisional cells, often presenting two stalk-like structures at opposite poles. These findings suggest that CaBP participates in the spatial and temporal regulation of cell differentiation events, possibly linking calcium fluxes to stalk and flagellum assembly. Recent studies on the biogenesis of the S-layer in *C. vibrioides* demonstrated that the assembly of this structure is coordinated with the cell cycle and depends on metal ions, especially Ca²⁺, for stabilization and anchoring to the envelope (Herdman et al. 2024). Thus, it is plausible that the absence of CaBP alters the periplasmic ionic balance and indirectly affects the integrity or distribution of these surface structures, resulting in the observed material consistent with adhesive matrix. In this context, CaBP may represent a component of a periplasmic calcium-sensing system associated with envelope homeostasis. Extracytoplasmic function (ECF) sigma factors frequently respond to signals originating in the cell envelope, including changes in membrane integrity, ion availability, and surface structure assembly (Mascher 2025). The periplasmic localization of CaBP together with its calcium-binding capacity raises the possibility that fluctuations in extracellular Ca²⁺ could be detected in the periplasm and transmitted to regulatory pathways controlling envelope remodeling and stress adaptation.

### Functional integration and evolutionary implications

To our knowledge, this study provides one of the first descriptions of a calcium-dependent regulatory circuit involving an EF-hand protein and an ECF sigma factor in *C. vibrioides*. Although the molecular component that transmits the CaBP signal to SigV remains to be identified, the model proposed here suggests that calcium sensing may represent an additional regulatory layer controlling growth and envelope-associated stress responses in *C. vibrioides*. More broadly, these results contribute to the emerging view that bacterial EF-hand proteins can function as environmental sensors integrating ionic signals into regulatory pathways that modulate bacterial physiology. In this context, CaBP may represent a previously unrecognized type of periplasmic EF-hand calcium sensor in Alphaproteobacteria, linking extracellular calcium fluctuations to regulatory circuits involved in envelope homeostasis and environmental adaptation. Future studies aimed at identifying the membrane-associated component connecting CaBP to SigV signaling will be essential to elucidate the molecular basis of calcium-dependent regulation in *C. vibrioides* and may reveal broader principles of bacterial calcium sensing.

## Supplementary Information

Below is the link to the electronic supplementary material.

Supplementary Material 1

Supplementary Material 2

Supplementary Material 3

## Data Availability

The data that support the findings of this study are available on requested from the corresponding author.
