# Supplementary figures and images for: A calcium-binding protein–sigV regulatory circuit modulates stress adaptation and persistence in Caulobacter vibrioides
Source: Arch Microbiol. 2026 May 13;208(8):389. doi: 10.1007/s00203-026-04894-7 (PMC13171946; doi:10.1007/s00203-026-04894-7)

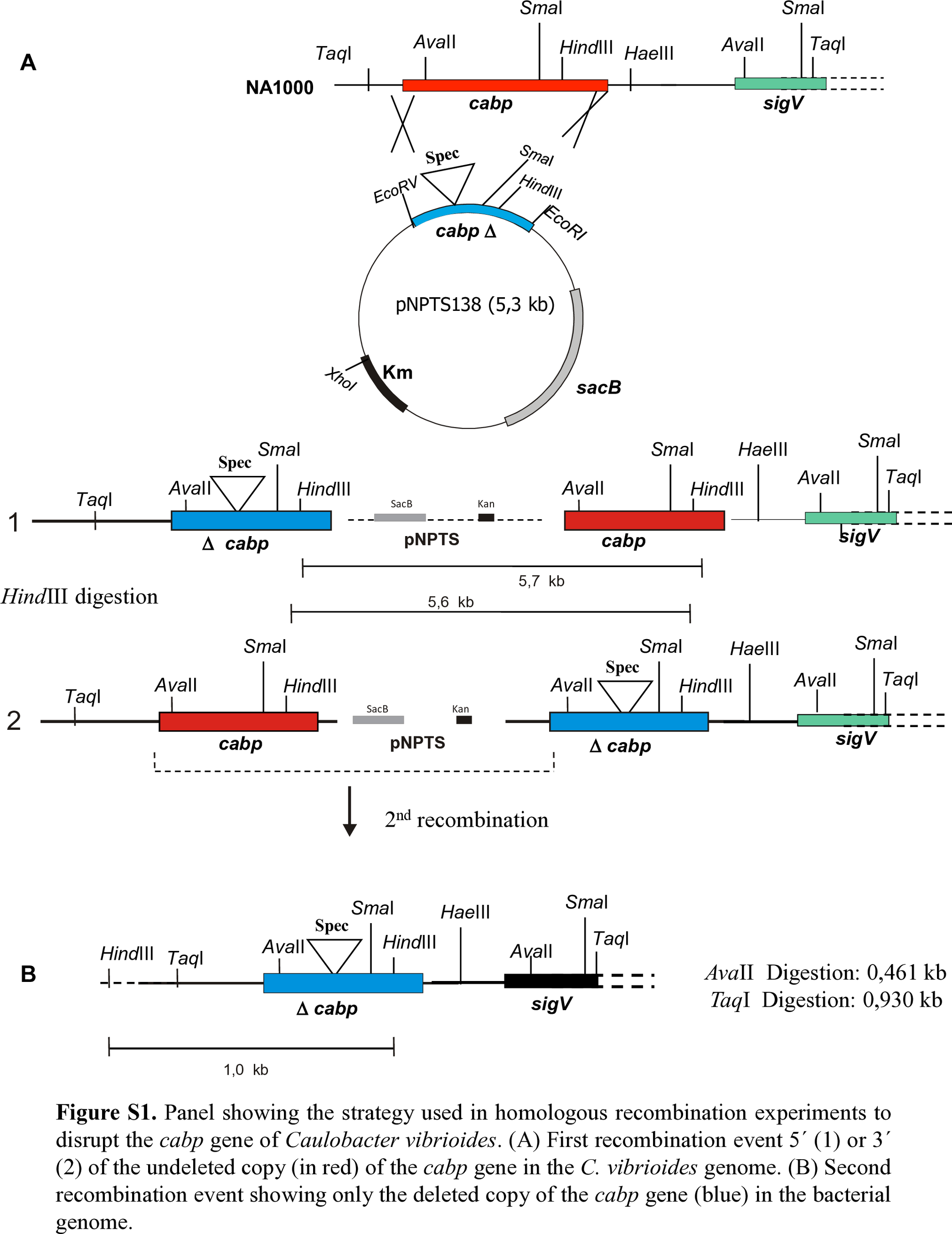

Supplement: Supplementary file 1 — Supplementary Material 1 [file 203_2026_4894_MOESM1_ESM.tif]

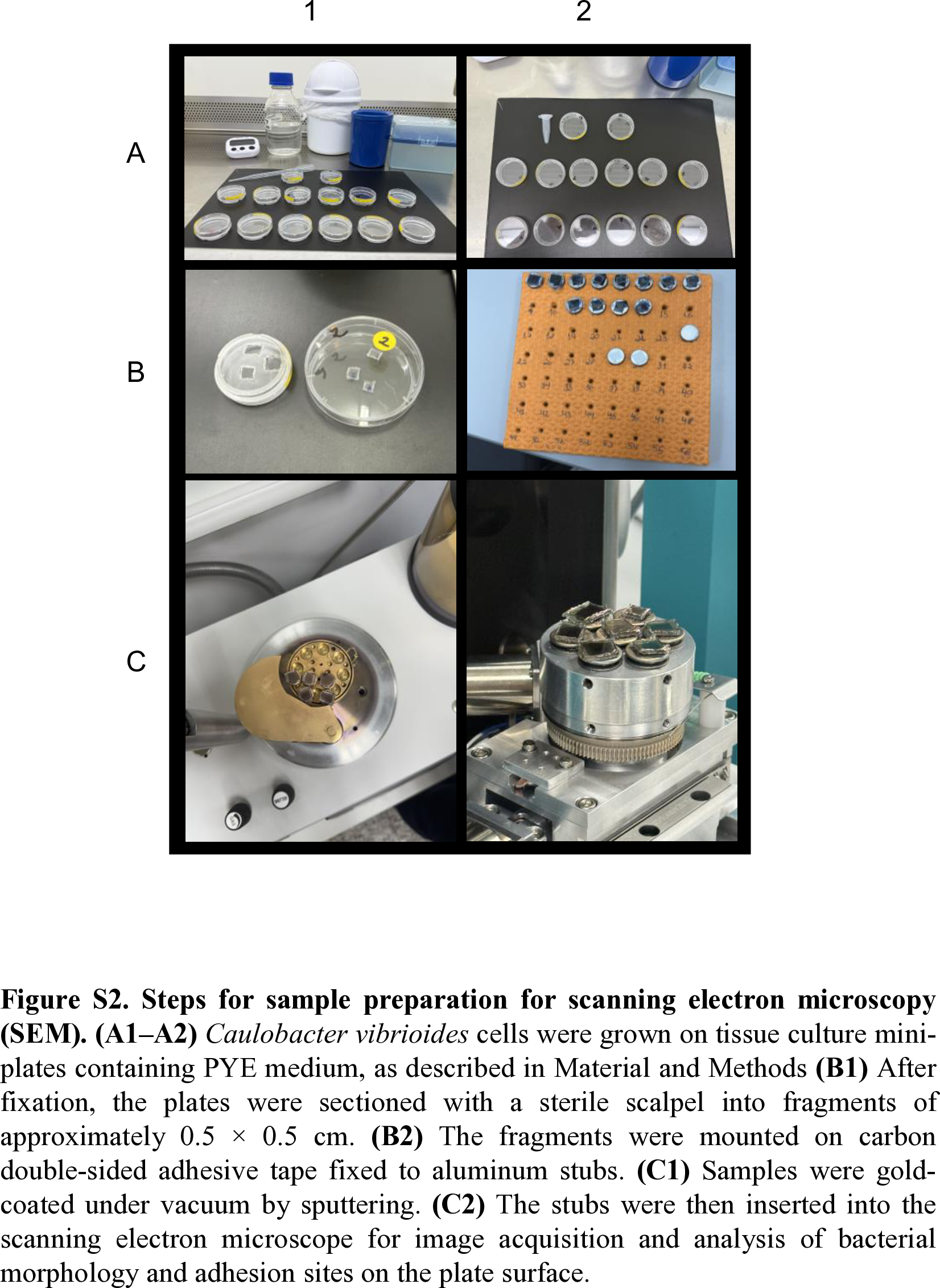

Supplement: Supplementary file 2 — Supplementary Material 2 [file 203_2026_4894_MOESM2_ESM.tif]

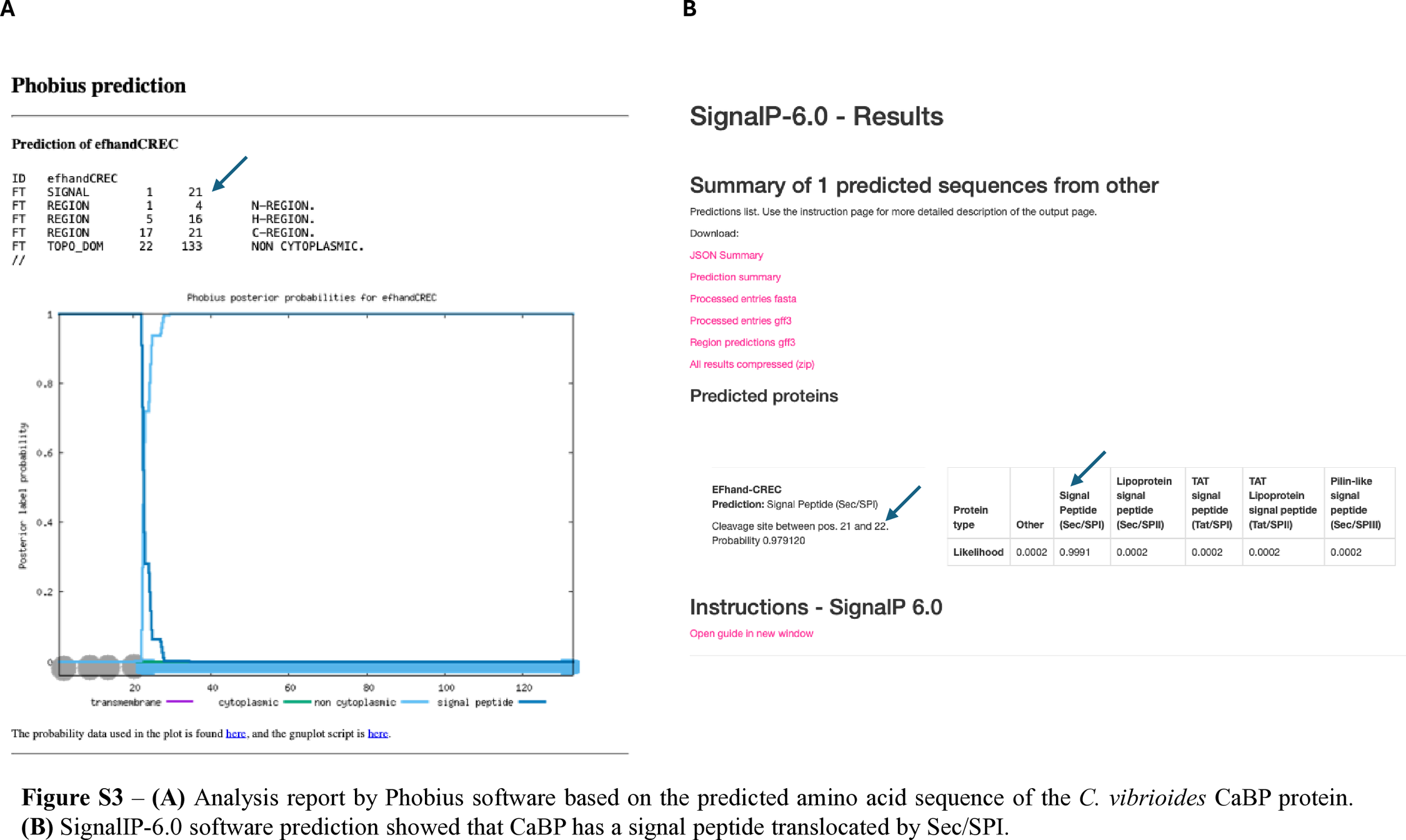

Supplement: Supplementary file 3 — Supplementary Material 3 [file 203_2026_4894_MOESM3_ESM.tif]
